# Optimized environmental prediction in smart buildings using Dynamic Greylag Goose algorithm and deep learning

**DOI:** 10.1038/s41598-026-41343-3

**Published:** 2026-03-28

**Authors:** Sayed Kenawy, Amel Ali Alhussan, Doaa Sami Khafaga, Ebrahim A. Mattar, Safaa Zaman, Marwa M. Eid

**Affiliations:** 1 Faculty of Artificial Intelligence, Delta University for Science and Technology, Mansoura, 11152 Egypt; 2 Department of Computer Sciences, College of Computer and Information Sciences, Princess Nourah bint Abdulrahman University, P.O. Box 84428, Riyadh 11671, Saudi Arabia; 3 College of Engineering, University of Bahrain, Zallaq, Bahrain; 4 Information sciences department ,College of life sciences, Kuwait University, Kuwait City, Kuwait

**Keywords:** Smart buildings, Environmental prediction, Long short-term memory, IoT sensor networks, Engineering, Mathematics and computing

## Abstract

The quick adoption of IoT technologies in smart buildings for monitoring the environment makes it possible to check environmental conditions frequently, but it also makes it challenging to process and analyze all the information regularly. This continuous data flow demands accurate forecasting models to support proactive environmental control in smart buildings. Although many studies focus on anomaly detection, fewer address high-accuracy environmental prediction enhanced by optimization. This paper addresses that gap by proposing a predictive framework combining feature selection and hyperparameter tuning. The framework integrates Dynamic Greylag Goose Optimization (DGGO) with a Long Short-Term Memory (LSTM) network. DGGO is applied in binary form for sensor feature selection to reduce input dimensionality, and again for tuning LSTM hyperparameters. This dual optimization improves the prediction of temperature, humidity, air quality, sound, and light. Experiments were conducted using a public IoT dataset from a smart building environment. Results show that DGGO-LSTM achieved the lowest Mean Squared Error (MSE) of 0.00119 and the highest Nash–Sutcliffe Efficiency (NSE) of 0.98247, outperforming GWO-LSTM (MSE = 0.00143), GGO-LSTM (0.00167), and WOA-LSTM (0.00190), corresponding to a 17–37% reduction in MSE. In addition, DGGO-LSTM demonstrated superior computational efficiency, reducing execution time to 145.32 s compared with 251 s for WOA (approximately 42% faster). These results confirm the framework’s strength in delivering robust, efficient, and high-accuracy environmental forecasting for intelligent building systems. The integration of deep learning with nature-inspired optimization presents a scalable approach for sustainable, data-driven control strategies.

## Introduction

The increasing use of IoT devices has changed industries and people’s lives by allowing constant acquisition, delivery, and analysis of huge volumes and biodiversity of real-time data. In homes, cars, industries, and hospitals, IoT devices are the order of the day, via which they create enormous data than could ever be anticipated^1^. This data provides a great opportunity to receive more value, improve the quality of activities, and solve problems through the application of knowledge. However, to bear the fruits of IoT, there is a major problem of handling and analyzing these voluminous contents^2^. Thus, IoT datasets are big, unstructured, and constantly changing, which necessitates the use of advanced data mining technique for getting useful knowledge from IoT data. In the field of time series forecasting which requires models that can capture the sequential nature of the data and complex, often nonlinear, interactions these challenges appear more stark^3^.

Big data generated by IoT is a high dimensional noisy and varying set which is based on the real time factors affecting the data set. These attributes make the task of accurately estimating various important parameters such as temperature particularly challenging^4^. The existence of temporal dependencies in which future states are dependent on the past values introduces another level of complexity requiring models than can understand these depictions. The right use of time-series forecasting is word-wide seen in such different fields as smart cities supporting energy utilization, environmental segments, and industrial automation with predictive maintenance and optimization^5^. For these requirements, novel computational paradigms are required, paradigms which not only capture complex data distributions but also maintain system stability under uncertainty, variability, and continuing growth of IoT data^6^.

AI and ML techniques have emerged as the primary means of getting around the challenges posed by IoT-produced data. They are flexible, data-based methods that offer enhanced predictive capability as the underlying system evolves^7^. Different types of ML include deep learning and forecasting techniques such as the LSTM network which render high performance for time-series data. LSTMs, in particular, are specifically developed for usage in cases when data is ordered in time, which is particularly compaction for capturing long-term dependencies and complex nonlinear relations characteristic for IoT data^8^.

One of the critical components of IoT analytics is responsible for deciphering useful information from IoT data sets. Sensitive to high dimensionality of the data, the IoT features may contain elements that might be irrelevant or redundant to the predictive mission^9^. However, if not well selected, these features result in poor performance, models that have over-fitted, and models that require steep processing power. Therefore, feature selection plays a crucial positive role in the process of dimensionality reduction and the optimization of predictive value as well as general modeling performance^10^.

Optimization algorithms have turned out to be promising solutions for the features selection and hyperparameters optimization problems^11^. These algorithms are based on concepts inspired by biological evolution and animal movement known to be the best when it comes to searching for the near-optimal solutions in such composite search space. The proposed metaheuristic method called the Dynamic Greylag Goose Optimization (DGGO) algorithm has proved outstanding in controlling exploration and exploitation^12^. This balance is important for a proper filtering of the subsets of features and to eliminate the local optimum. As integrated with LSTM models, DGGO allows for the enhancement of the forecast accuracy and reduced computational expenses since it is particularly useful in the IoT high-dimensional data environment^13,14^.

Feature selection is not just a precursor but can make or mar a predictive framework. It also improves the quantity and quality of the data that is fed into machine learning model since it simplifies the model by removing redundant data set dimensions^15^. The approaches such as DGGO applied in selecting the features guarantee that those features give high prediction power to models like LSTM. Such integration between feature selection and model construction is particularly critical in IoT settings since data layouts and amounts are complex^16^.

**The main objectives of this paper are:**

1. To develop an intelligent and adaptive framework for environmental prediction and monitoring in smart buildings, using advanced deep learning and optimization techniques, with a focus on enhancing indoor environmental quality and operational reliability.
2. To introduce and apply the Dynamic Greylag Goose Optimization (DGGO) algorithm as a bio-inspired approach for optimizing high-dimensional IoT sensor data, enabling efficient and scalable feature selection for building control systems.
3. To integrate Long Short-Term Memory (LSTM) networks for time-series analysis of indoor environmental parameters, capturing complex temporal dynamics such as fluctuations in temperature, humidity, and air quality to support responsive building automation systems.
4. To evaluate the proposed DGGO-LSTM prediction model against other optimization-based forecasting techniques, establishing its superiority for smart building applications related to energy efficiency, occupant comfort, and system resilience.

The remainder of this article proceeds as follows. Section “Literature review” surveys prior work on IoT-based time-series modeling, with emphasis on environmental prediction, feature selection, and nature-inspired optimization. Section "Materials and methods" details the proposed methodology, describing the dataset, preprocessing pipeline, and the DGGO–LSTM integration for binary feature selection and hyperparameter tuning. Section “Experimental results” reports experimental results, including performance evaluations using MSE and NSE, and benchmarks against alternative deep models and optimizers. Section “Discussion” concludes with key findings, limitations, and avenues for future research on scalable, prediction-focused, and optimization-driven frameworks for smart buildings.

## Literature review

The international community widely acknowledges the ongoing changes in Earth’s climate, necessitating collaborative global efforts to mitigate its impacts. In the view of^17^, the European Union (EU) has taken a proactive stance by launching initiatives aimed at reducing greenhouse gas emissions and enhancing energy efficiency, particularly through advanced building energy management systems. Traditional buildings often rely on households to manually monitor and control Heating, Ventilation, and Air Conditioning (HVAC) systems, leading to inefficient energy usage, such as devices being left on unintentionally. Smart buildings now automate these processes, dynamically adjusting HVAC systems based on user preferences to enhance satisfaction while optimizing energy consumption. An analysis comparing 36 machine learning algorithms for forecasting indoor temperature in smart buildings demonstrated that the ExtraTrees regressor outperformed other methods in both accuracy (0.97%) and performance (0.058%) based on experiments using real data.

Optimizing heating, ventilation, and air conditioning systems for comfort and energy efficiency remains a challenge, particularly during crises like COVID-19. As reported by^18^, predictive accuracy often declines during repeated forecasting, limiting the effectiveness of current methods. Advanced mechanisms were presented for analyzing sensor data and applying a new approach for a time-series forecasting based on adaptive learning. Garnered models showed 17.4% of energy saving and 16.9% of comfort improvement. It describes what it means to forecast repeatedly, and how new solutions help to increase effectiveness and make occupants of smart buildings more comfortable.

Accurately forecasting meteorological parameters such as air temperature and humidity is vital for effective air quality management. As evidenced in the study by^19^, various machine learning models, including gradient boosting, random forest, linear regression, and artificial neural networks, were evaluated for their ability to predict these parameters. Using 24 years of daily data from the Malaysia Meteorological Department, the multi-layer perceptron neural network performed best for daily temperature and humidity predictions, achieving correlation coefficients of 0.7132 and 0.633, respectively. For monthly predictions, the same model excelled for temperature with a correlation of 0.8462, while the radial basis function neural network outperformed others for humidity with a correlation of 0.7113. Validation with unseen data confirmed that both neural network models can effectively predict daily and monthly values with reasonable accuracy, demonstrating their potential for practical application in meteorological forecasting.

A comprehensive analysis of 500 scientific articles published since 2018 was conducted to explore the application of machine learning techniques in climate science and numerical weather prediction. As demonstrated by^20^, the study identified key topics in these areas, including photovoltaic and wind energy, atmospheric processes in weather prediction, and parametrizations, extreme events, and climate change in climate research. Commonly analyzed meteorological variables included wind, precipitation, temperature, pressure, and radiation, with popular methods such as deep learning, random forest, artificial neural networks, support vector machines, and extreme gradient boosting. The study also highlighted countries leading research efforts, including China, the USA, Australia, India, and Germany. The findings suggest that machine learning will play a central role in advancing weather forecasting and climate modeling, indicating significant potential for future developments in these fields.

High temperatures reduce concrete strength by altering material properties, making it challenging to achieve desired compressive strength. As reported by^21^, supervised machine learning techniques such as decision trees, neural networks, bagging, and gradient boosting were used to predict concrete strength under high temperatures using nine input variables, including temperature and material composition. Gradient boosting and bagging showed the highest accuracy, with $R^2$ values of 0.90 and 0.88, outperforming decision trees and neural networks. Statistical metrics and sensitivity analyses highlighted the effectiveness of ensemble methods, confirming their potential to enhance predictive accuracy in high-temperature concrete applications.

The Internet of Things (IoT) enables billions of devices to generate data by sensing and interacting with their environments, producing structured, semi-structured, or unstructured data in real-time or batch formats. As outlined by^22^, The use of open-source tools such as Apache Spark and Kafka for developing a predictive analytic solution for temperature time series data was put forward. The descriptive methods included ARIMA and SARIMA while the predictive methods included LSTM and the novel CNN-LSTM model. It was found that litter per liter deep learning models particularly CNN LSTM outperformed traditional methods in terms of error rates and accuracy. Thus, remarkably, the hybrid CNN-LSTM model showed the highest accuracy in temperature prediction, which proves its efficacy in the given tasks.

Firefighters face significant risks when navigating burning structures, including extreme heat, toxic gases, and structural instability. As highlighted by^23^, this research advocates the use of deep learning technique alongside predictive modelling to improve safety aspect and decision making in such cases. The danger level is identified using an unsupervised deep learning classifier, temperature trends are predicted by a random forest regressor and an autoregressive integrated moving average (ARIMA) model. The models describe temperature fluctuations on one or more levels above ground level, which, at 2.6 meters, are enough to compromise structural stability. Though the autoencoder artificial neural network yielded decent classification accuracy of 0.869, the ARIMA model did a good job in the temporal forecasting of temperature necessary for the firefighting activities. The results of this paper show that wireless sensors and other methods of advanced analytics can be used for enhancing situation awareness and safety of firefighters.

Accurate and efficient temperature prediction algorithms are increasingly essential for maintaining optimal charging and operational conditions in electric vehicles. As outlined by^24^, this study designed a real time temperature dataset with the help of Internet of Things and the sensor based hardware mounted on a vehicle body. The collected data was then analyzed and used to develop supervised regression models to predict the temperatures in future with high accuracy. Statistical error such as Mean Absolute Error, Mean Squared Error, Root Mean Squared Error, and Coefficient of Determination scores were used in this study to empirically compare the predictions to the actual recorded values. This does not depend on parameters to capture high temperatures and generate an alarm to advise the drivers to change their temperature settings.

Temperature data is fundamental for meteorological, hydrological, and climate studies, and the completeness of this data is critical for research reliability. As outlined by^25^, this study compared machine learning methods, including support vector machines, adaptive neuro-fuzzy inference systems, and decision trees, to fill missing air temperature data. Using 50 years of monthly average temperature data (1968–2017), models were trained and tested with an 80/20% split. Neighboring stations with high correlations were used as inputs for prediction. The adaptive neuro-fuzzy inference system, configured with four subsets, a triangular membership function, and 300 iterations, outperformed others, showing the lowest errors and highest determination coefficient. The study recommends using adaptive neuro-fuzzy inference systems for estimating monthly air temperatures in northeastern Turkey and other semi-arid regions.

Smart buildings increasingly integrate technologies like artificial intelligence, the Internet of Things, and cloud computing to enhance comfort and reduce energy waste. As outlined by^26^, current heating, ventilation, and air conditioning system control strategies rely on multiple sensors to monitor indoor environment variables, but extensive sensor networks can be costly and complex to manage. This research addresses the issue by applying machine learning to estimate unmeasured variables using fewer sensors. Using six months of data from a smart building in Japan, extreme gradient boosting models were developed to estimate room temperature, humidity, and CO2 levels. The models achieved high accuracy, with root mean squared errors of 0.3 degrees for temperature, 2.6% for humidity, and 26.25 ppm for CO2. These results demonstrate the potential to optimize HVAC systems while reducing sensor requirements, offering a cost-effective solution for smart building management.

Beyond traditional forecasting approaches, recent work has emphasized the role of causal reasoning and structured representations in improving model robustness and interpretability. For example, Lu et al. formulated image super-resolution within the framework of structural causal models, explicitly modeling degradation mechanisms rather than treating the task as a black-box process^27^. Their approach introduces counterfactual learning and adaptive intervention mechanisms to isolate and analyze degradation factors such as blur and noise, producing invariant representations capable of maintaining reconstruction quality across complex degradation scenarios. Experimental results demonstrated consistent improvements over state-of-the-art methods while providing interpretable and disentangled causal feature representations.

In industrial visual inspection, deep learning–based defect detection has advanced by removing non-differentiable components that hinder full end-to-end training. Lu et al. proposed a differentiable non-maximum suppression (NMS) mechanism based on Sinkhorn bipartite matching, ensuring continuity of gradient flow throughout the detection pipeline^28^. Their formulation jointly considers proposal quality, spatial relations, and feature similarity, while an entropy-constrained mask refinement step enhances localization accuracy under irregular defect boundary conditions. Evaluations on the Tianchi benchmark confirmed improved robustness, precision, and processing efficiency, validating the model for real-world industrial applications.

Further improvements in defect interpretability and class imbalance were addressed by Lu et al. through a reconstruction-driven spatial cloze strategy^29^. Their method frames defect detection as an image completion task, training a restoration network to reconstruct removed local regions of the defective image. A progressive attention fusion mechanism then combines the reconstructed defect-free result with the original input to highlight abnormal regions through image comparison. Experimental results showed superior performance across 92% of defect categories, demonstrating strong interpretability and generalization, particularly for rare defect classes.

In the field of multivariate sensor analysis, researchers have increasingly focused on learning inter-variable dependencies to support anomaly detection and fault diagnosis. Deng and Hooi presented a graph neural network–based approach capable of jointly learning temporal dynamics and latent relational structures between sensor streams^30^. Their model includes a data-driven structure learning module and an attention-based anomaly explanation mechanism, enabling users to identify not only when anomalies occur but also which variables contributed to them. Experimental validation on real industrial sensor datasets confirmed improved detection accuracy and interpretability compared to conventional deep learning baselines.

Scalable unsupervised anomaly detection for large multivariate systems has been introduced through adversarial autoencoder architectures. Audibert et al. proposed the USAD framework, which learns compact latent representations of normal patterns and isolates anomalous sequences by leveraging adversarial learning dynamics^31^. This method was validated on five public datasets as well as proprietary large-scale IT infrastructure data, demonstrating competitive anomaly detection performance, fast training, and robustness to high-dimensional sensor environments, making it highly suitable for IoT and cloud-integrated monitoring platforms.

Recent advances in deep learning have also introduced attention-based and causality-aware models that address limitations of purely black-box learning in complex prediction settings. In the time-series domain, Informer was proposed as an efficient transformer architecture for long sequence time-series forecasting, introducing a probabilistic sparse self-attention mechanism and a distillation strategy to reduce computational cost while preserving long-range dependency modeling capability^32^. Similarly, the Temporal Fusion Transformer (TFT) combines recurrent processing with interpretable self-attention and gating mechanisms to support multi-horizon forecasting with heterogeneous inputs (e.g., static covariates and known future variables), while providing interpretability regarding temporal dynamics and feature relevance^33^. Beyond forecasting, causality-driven deep learning has been explored to improve robustness and interpretability under complex degradation and distribution shifts; for example, CausalSR formulates super-resolution through structural causal modeling and counterfactual reasoning to obtain invariant representations and interpretable insights^27^. Collectively, these studies highlight the growing importance of efficient attention mechanisms and causal reasoning for handling long-range dependencies and improving interpretability in modern predictive systems.

## Materials and methods

The following section will look at the kind of methodology that was used in developing a strong framework for predicting environmental parameters within smart building environment IoT sensor data. It uses data acquisition, data preprocessing, feature selection and reduction, topology optimization, and a deep learning algorithm to accurately forecast multiple sensor variables within the time-series dataset rather than detecting anomalies.

**Fig. 1:**
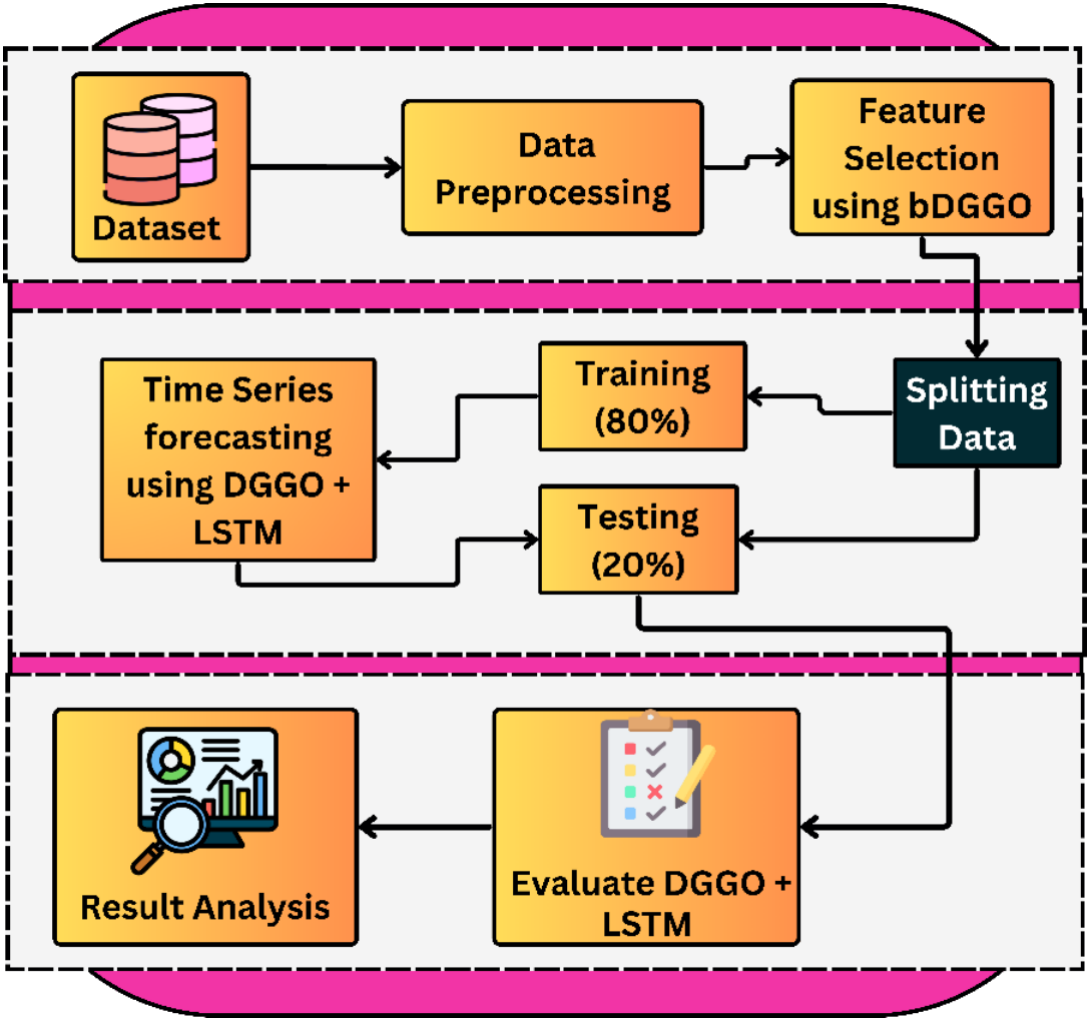
Architecture of the Proposed DGGO-LSTM environmental prediction framework.

Figure 1 illustrates the proposed systems-level architecture, which follows a comprehensive pipeline from raw IoT sensor data acquisition at the edge to environmental parameter prediction. The framework encompasses data preprocessing, feature selection, model training, and evaluation, with each component designed to enhance performance and real-world applicability in intelligent environments. The system leverages the Dynamic Greylag Goose Optimization (DGGO) algorithm, which incorporates the adaptive behavioral characteristics of greylag geese, to perform optimal feature selection and hyperparameter tuning for the LSTM network. By integrating bio-inspired optimization with deep learning, the framework effectively captures temporal patterns, handles sensor data variability, and achieves robust environmental forecasting in indoor environment monitoring systems.

### Data analysis

The data utilized in this work was gathered from a smart building context and encompasses time series data that was generated through a set of Grove sensors. These are basically used to get the key environmental metrics including temperature, humidity, light intensity, air quality and noise levels. The node holds the Grove – Temperature & Humidity Sensor (High-Accuracy & Mini) v1.0; Grove – Light Sensor; Grove – Loudness Sensor; Grove – Air Quality Sensor v1.3. For communication and data transmission, the system employs Digi XBee 3 Zigbee 3 RF Module and Grove – UART WiFi V2. Altogether, this chain of sensors ensures a constant and multivariate approach to monitor and perceive the changes within and across indoor environments.

**Fig. 2:**
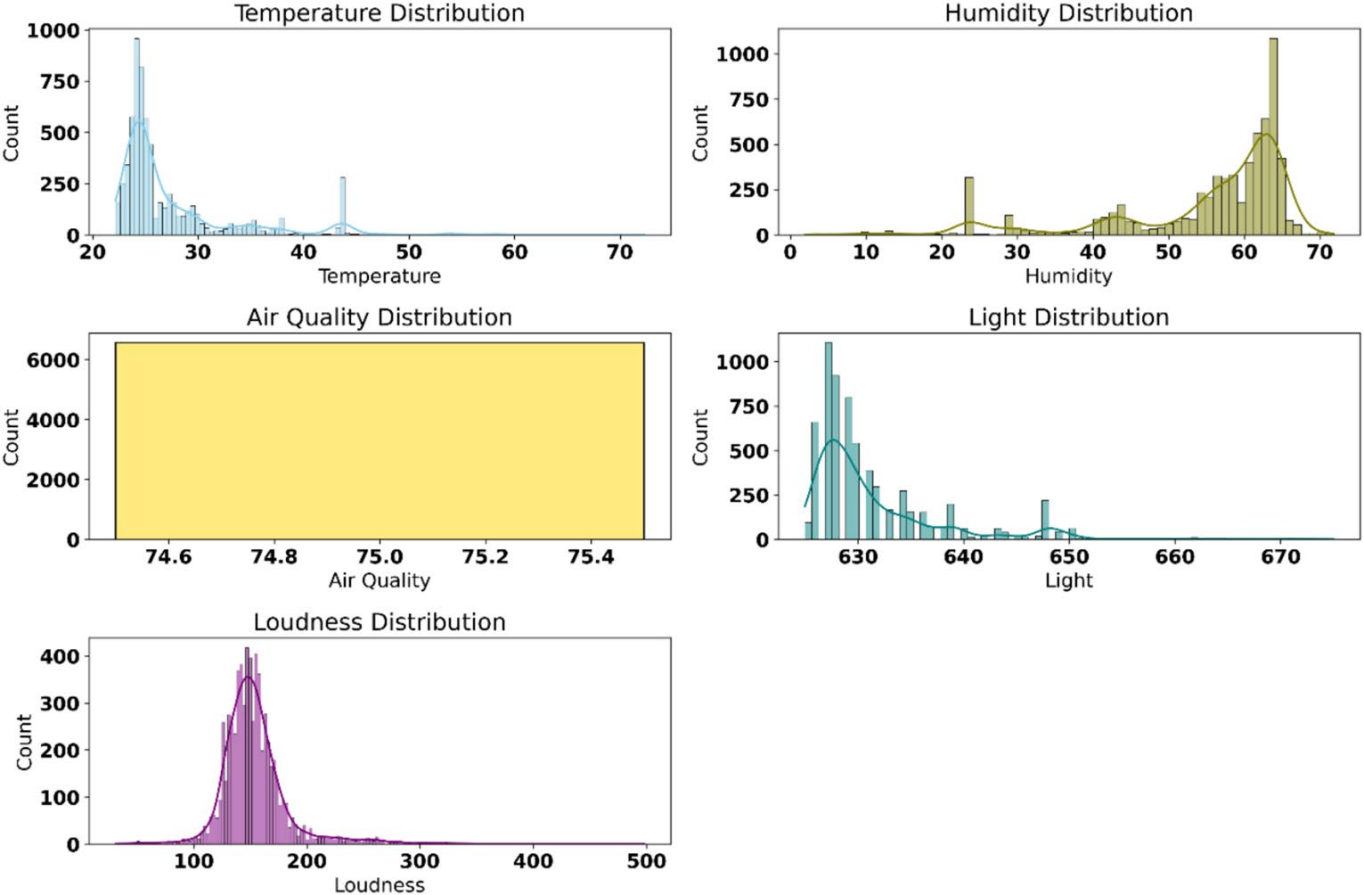
Distributions of environmental parameters collected via IoT sensors.

Figure 2 shows the distribution of environmental parameters measured using the IoT pipeline. The top left plot highlights the temperature distribution, which is skewed towards lower temperatures, with a few higher values indicating variability. The top right plot illustrates the humidity distribution, showcasing a bimodal trend with peaks at mid-range and higher humidity levels. The air quality distribution in the middle-left plot exhibits an almost flat distribution, suggesting consistent measurements over the range observed. The middle right plot of light distribution demonstrates a right-skewed pattern with most data concentrated at lower levels, tapering off gradually. Finally, the bottom plot reveals a bell-shaped curve for loudness distribution, indicating a concentrated range of noise levels with minor outliers.

**Fig. 3:**
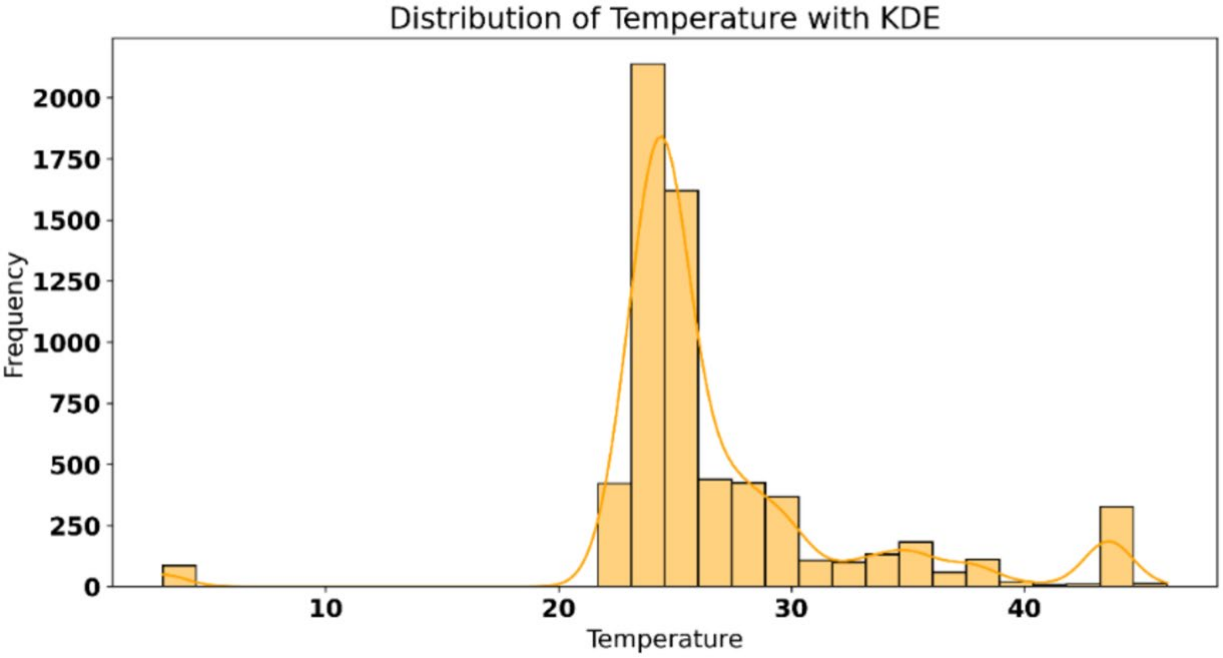
Temperature distribution with KDE analysis.

Figure 3 shows the distribution of temperature values observed in the dataset, accompanied by a Kernel Density Estimate (KDE) curve for smooth visualization of underlying trends. The histogram reveals a prominent peak in the temperature range that represents the most frequent readings. The KDE curve aligns closely with the data, highlighting the skewness towards higher temperatures while capturing minor variations at extreme ends. A secondary rise at higher temperatures may indicate sporadic occurrences of elevated conditions. The distribution is asymmetric, with a long tail towards lower and higher temperatures, suggesting variability across observations.

**Fig. 4:**
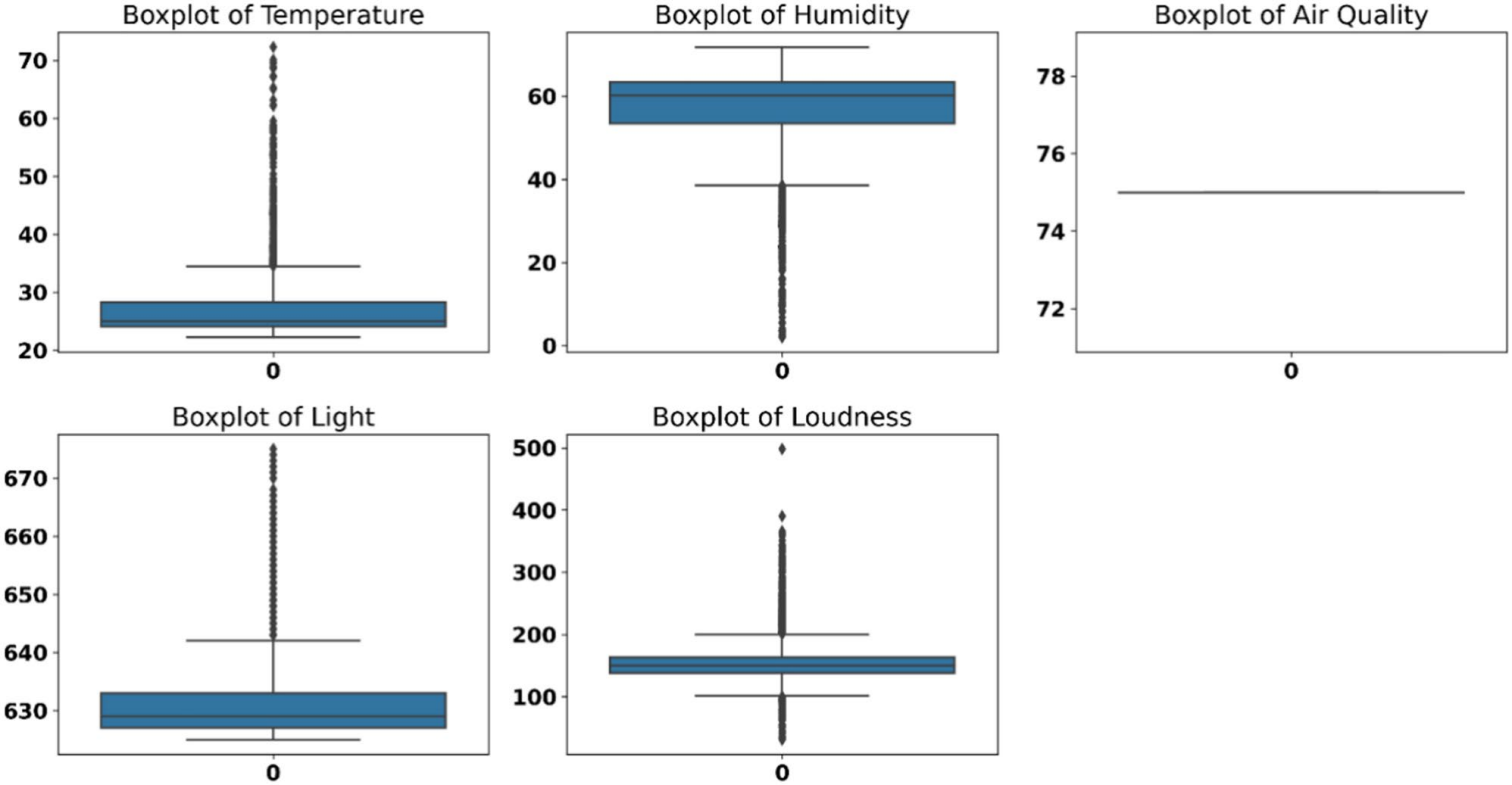
Boxplots of environmental parameters.

Figure 4 shows the boxplots for various environmental parameters, offering a concise view of their central tendency and variability. The boxplot of temperature (top left) indicates a high number of outliers on the upper end, with the interquartile range capturing most of the data below a moderate level.

**Fig. 5:**
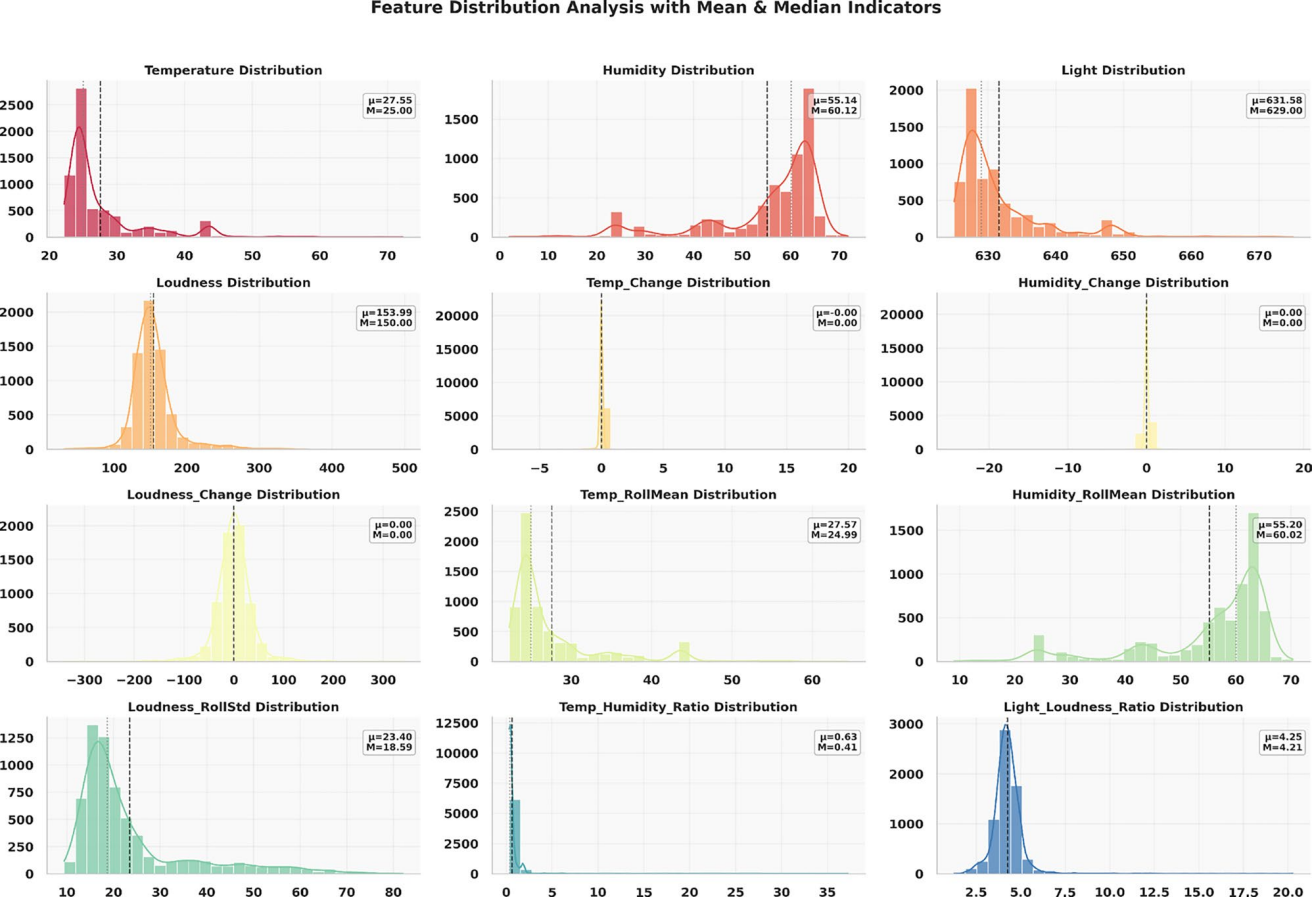
Feature distribution analysis of environmental sensor data.

Figure 5 illustrates the distributions of several environmental and derived features. The plots show the statistical behavior of different features like Temperature, Humidity and Light. The distribution of Loudness is also visualized. Also included are the distributions of the ratio between temperature and humidity, and the rate of change of Temperature, Loudness and Humidity. Some features exhibit relatively narrow distributions centered around their mean and median, such as Temperature, while others, like Loudness, display broader distributions indicating higher variability. Rolling mean distribution of temperature is also provided. Also standard deviation of Loudness distribution is visualized.

Since the dataset does not contain validated anomaly ground truth, the following visualization presents an exploratory comparison between samples flagged by the derived deviation indicator Is_Anomaly_t rather than true normal or anomalous states.

**Fig. 6:**
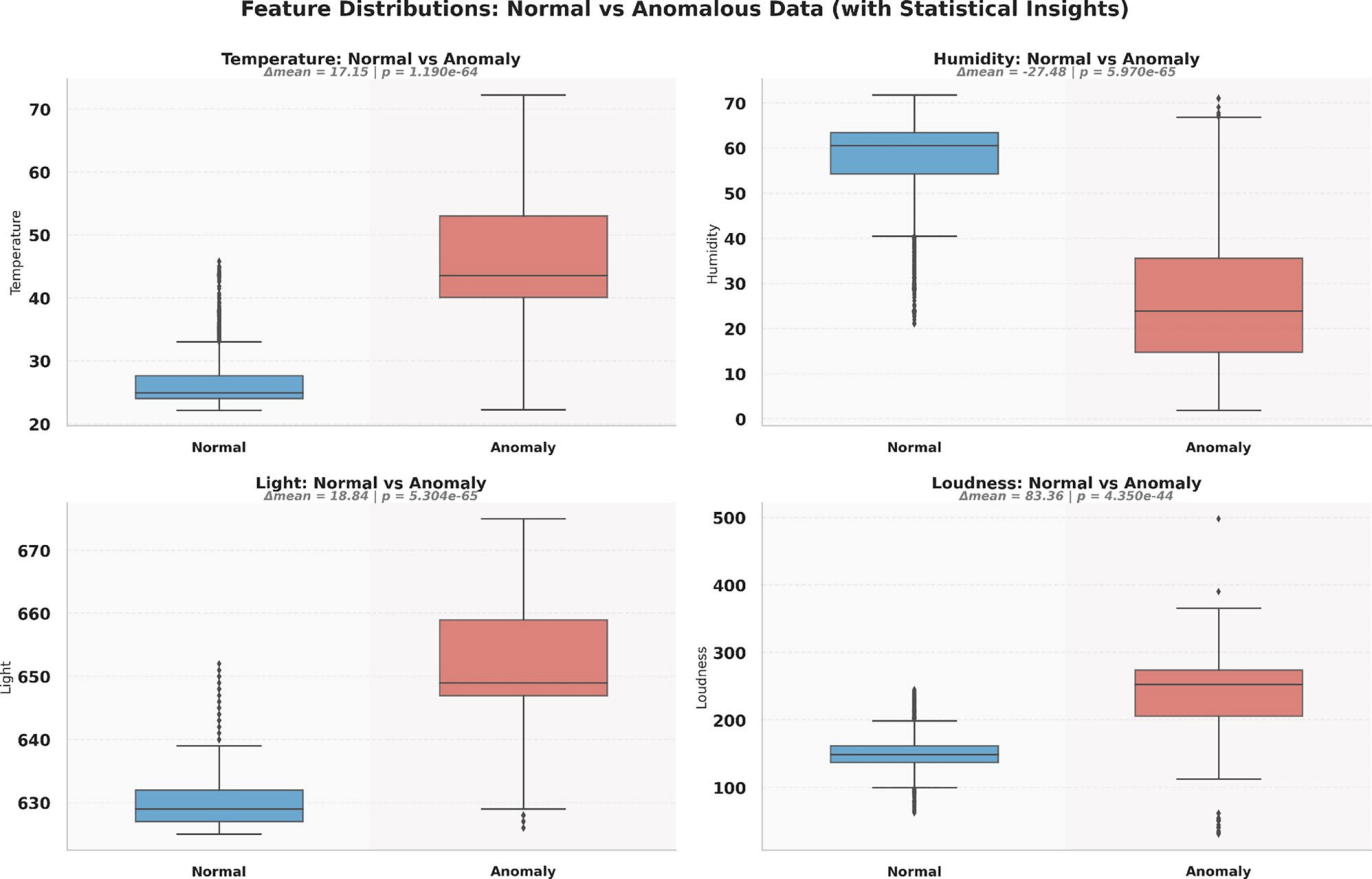
Distribution of normal versus high-deviation observations derived from threshold-based sensor deviations.

Figure 6 shows the distributions of different features. The statistical distribution plots reveal variation patterns across the recorded environmental parameters rather than separating anomalous versus normal conditions. The temperature values exhibit a relatively higher concentration in the upper range compared to the remaining features, indicating stronger variability within this parameter. Light data show discretized or fixed step values, suggesting sensor quantization or threshold?based recording. Loudness displays a noticeably wider spread across its value range, reflecting higher dynamic fluctuation compared to other sensor variables.

### Preprocessing pipeline

To ensure the robustness and predictive accuracy of the proposed framework, an extensive preprocessing pipeline was applied to the raw environmental dataset. This pipeline encompassed data cleaning, timestamp parsing, feature engineering, and data normalization. Outlier detection was not included, as the environmental sensor data was assumed to be reliable following prior calibration and filtering. First, missing values were handled through linear interpolation, which leverages adjacent timestamps to estimate missing sensor readings. This is particularly effective in time-series datasets, where values typically evolve gradually. All timestamp entries were converted to a consistent datetime format to facilitate temporal analysis and alignment. The dataset was then augmented through an intensive feature engineering phase to extract informative representations of the environmental patterns. Time-based features such as Hour, Day, and Weekday were derived from the timestamp to capture diurnal and weekly cycles in environmental behavior. These temporal features enabled the model to learn periodic patterns commonly exhibited by natural phenomena.

Next, change-based features were computed by differencing each sensor reading with its immediate past value, such as Temp_Change_t, Humidity_Change_t, and Loudness_Change_t. These capture sudden deviations and transient dynamics that could indicate abnormal system behavior or sensor drift.

To enhance the model’s understanding of local trends and reduce high-frequency noise, rolling statistics were introduced. These included rolling means for temperature and humidity, and rolling standard deviation for loudness, computed over a fixed window w. These rolling features provide smoothed versions of sensor behavior, which help in stabilizing model learning.

In addition, interaction-based features were incorporated to capture interdependencies between environmental variables. For example, Temp_Humidity_Ratio and Light_Loudness_Ratio were constructed to reflect cross-variable dynamics. These ratios are crucial in anomaly detection tasks, where unusual combinations of features may be more informative than individual variable anomalies.

A deviation-based metric was also implemented by computing the absolute difference between the current temperature and its rolling mean. This value was then thresholded to produce a binary feature, Is_Anomaly_t, that flags significant deviations. This anomaly indicator was not used as a ground truth label but as an auxiliary signal in the learning process, and serves only as a derived deviation flag for feature enrichment and exploratory distribution analysis rather than supervised anomaly classification.

Lastly, feature scaling was performed using min-max normalization across all variables to ensure uniform value ranges. This step is essential for convergence in gradient-based models such as LSTM.

The final feature set comprised 13 engineered variables:Hour, Day, Weekday, Temp_Change, Humidity_Change, Loudness_Change, Temp_RollMean, Humidity_RollMean, Loudness_RollStd, Temp_Humidity_Ratio, Light_Loudness_Ratio, Deviation, Is_Anomaly. This transformation increased the dataset’s expressiveness without introducing additional dimensionality via external sources.

### Dynamic Greylag Goose Optimization (DGGO)

Geese are social and dynamic bird that are somehow adaptive and that makes them to be cooperative. They develop long-term partnerships with their spouses, which reflects loyalty by guarding them during sickness or disability. Getting to a more interesting aspect, it could move to isolation, and remain a lone goose for the rest of its duration, a factor which depicts a high level of loyalty in a creature that most of them could not offer for even long-time companions. In the breeding period, birds also perform any breeding activities, and males will defend the eggs or nests very actively while females incubate them. Some birds of the goose species may exhibit the behavioral response known as philopatry, where they return to a nest, if suitable environmental conditions are present.

**Fig. 7:**
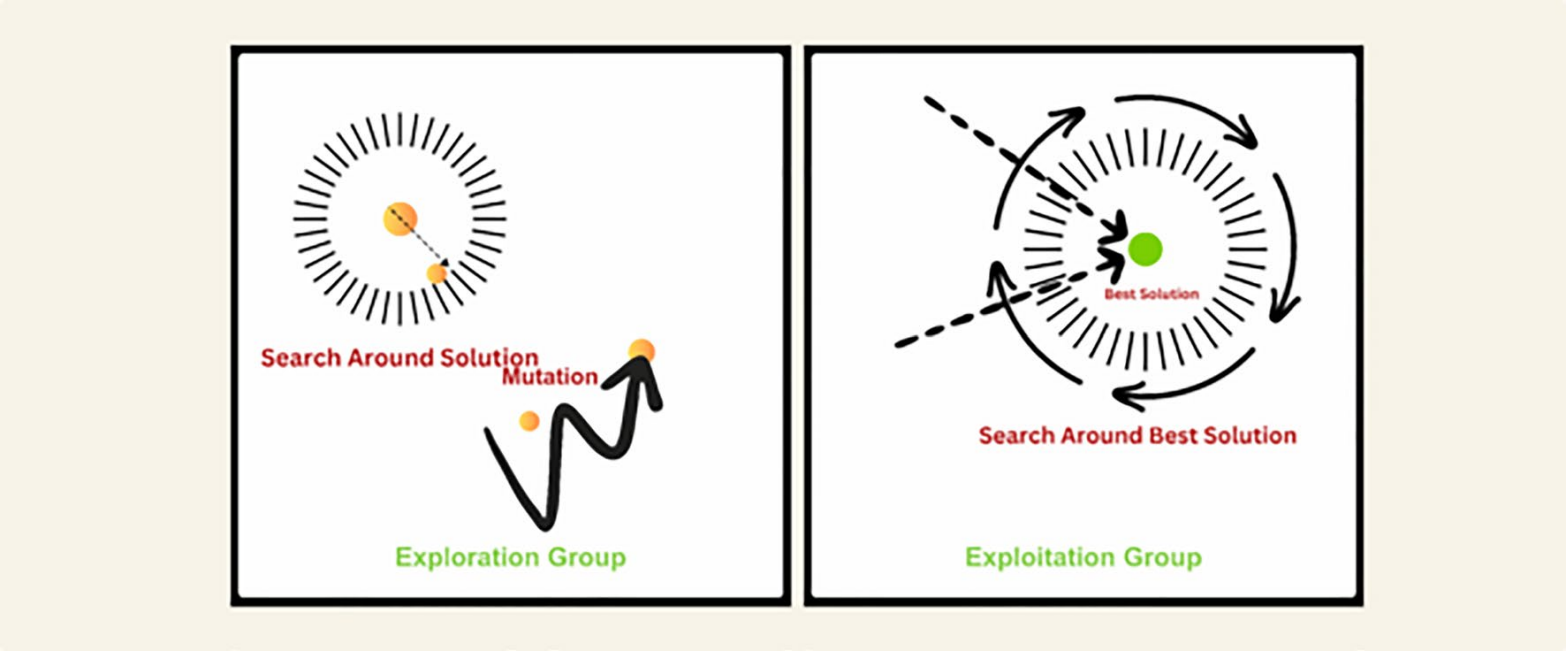
Exploration and exploitation of Grey Goose Optimization.

This denotes the commitment that they have towards ensuring that the future generation will not be exposed to vice. In larger gatherings where many geese come together, the commonly used term is a gaggles. In these groups, rotation is used and persons who are on their shift are the ones guarding predators while the rest feed. It is like the way sailors on a ship are packed working as a team where all members are watching each other. Watching the well-being of geese it is possible to notice the actions of some of them: protecting the injured zones, and the activity can be stated as typical for the brood. This means so is the migration of geese. They fly in large groups throughout thousands of kilometers. This is because in flying formation, they assume V-shape formation whereby this cuts down on the drag and as such, energy is conserved in longer flights. They have also a joint memory and navigation system; their movement is profoundly experienced which includes recognized reference points and sky indicators. The Greylag Goose Optimization (GGO) algorithm draws this concept of dynamic behavior of geese mentioned above. It starts by generating random solutions: each of the terms refers to a potential solution to a specific problem. These are then divided into exploration and exploitation parties, denoted in our implementation by two explicit sub-populations of sizes $(n_1,n_2)$, which are processed using distinct movement operators within each iteration, leading to an efficient search for solution space and preventing the algorithm from being trapped in local optima as illustrated in Fig. 7.

However, the proposed Dynamic Greylag Goose Optimization (DGGO) extends the standard GGO by introducing an operator-switching and time-varying control mechanism that makes the search process dynamic rather than static. In DGGO, key parameters such as the weights $(w_{1}, w_{2}, w_{3})$ and the factor (*z*) are updated throughout the iterations instead of remaining constant. In particular, the parameter *z* is explicitly updated at each iteration by $z = 1-\left( \frac{t}{t_{max}}\right) ^2$, which gradually shifts the search from exploration-oriented movements to more refined exploitation. Moreover, DGGO employs multiple complementary update operators and selects among them based on iteration parity $(t \bmod 2)$ and stochastic conditions (e.g., $r_3$ and |*A*|), enabling different behaviors to be activated at different stages of the search. This explicit operator-selection structure, together with the time-varying *z*, constitutes the main implementation difference captured in Algorithm 1.

#### Exploration operation

The exploration operation within the GGO algorithm’s meta-heuristic strategy is derived from the objective of identifying the most suitable areas within the search space as well as the prevention of getting stuck to a local optimum by migrating towards the global optimum. This is done by changing positions of the agents in a repetitive sequence and applying certain equations. Another essential equation in the GGO algorithm to update the positions of the agents came up as follows:

1 $$\begin{aligned} \textbf{X}(t+1) = \textbf{X}^{*}(t) - \textbf{A} \cdot \left| \textbf{C} \cdot \textbf{X}^{*}(t) - \textbf{X}(t) \right| \end{aligned}$$

Another equation used to update agent positions involves utilizing three random search agents, named $\textbf{X}_{\text {Paddle 1}}$, $\textbf{X}_{\text {Paddle 2}}$, and $\textbf{X}_{\text {Paddle 3}}$, and is expressed as:

2 $$\begin{aligned} \textbf{X}(t+1) = w_{1} \cdot \textbf{X}_{\text {Paddle 1}} + z \cdot w_{2} \cdot \left( \textbf{X}_{\text {Paddle 2}} - \textbf{X}_{\text {Paddle 3}} \right) + (1-z) \cdot w_{3} \cdot \left( \textbf{X} - \textbf{X}_{\text {Paddle 1}} \right) \end{aligned}$$

Where $w_{1}, w_{2}, w_{3}$ are updated within $[0,2]$, and $z$ is calculated at each iteration based on iteration number. This gradual change in *z* controls the transition from global search (large *z*) to precise local refinement (small *z*), and is one of the dynamic control mechanisms that differentiate DGGO from standard GGO. As implemented in Algorithm 1, the exploration sub-population applies these operators conditionally based on $(t \bmod 2)$, $r_3$, and |*A*|, which allows the algorithm to alternate between leader-guided exploration, paddle-based differential movement, and oscillatory exploration patterns.

Let $X_{\text {Paddle}}^{1,2,3}$ represent the set of candidate solutions (positions) for the agent in the search space. The term “Paddle” refers to a set of positions that guide the optimization process.

Additionally, another updating process involves adjusting vector values based on certain conditions, and is expressed as:

3 $$\begin{aligned} \textbf{X}(t+1) = w_{4} \cdot \left| \textbf{X}^{*}(t) - \textbf{X}(t) \right| \cdot e^{bl} \cdot \cos (2\pi l) + \left[ 2w_{1}(r_{4} + r_{5}) \right] \cdot \textbf{X}^{*}(t) \end{aligned}$$

where $b$ is a constant, $l$ is a random value in $[-1,1]$. The $w_{4}$ parameter is updating in $[0,2]$, while $r_{4}$ and $r_{5}$ are updating in $[0,1]$. These controlled oscillatory movements allow DGGO to maintain exploration ability even in later stages, reducing the risk of premature convergence. In Algorithm 1, we define the weights $w_4$ and the random factors $r_4$ and $r_5$ used for balancing exploration and exploitation during the search process.

#### Exploitation operation

During the exploitation phase, the optimal locations of the Greylag Goose Optimization (GGO) algorithm aim at enhancing the quality of current solutions. The GGO sorts the solutions with the best fitness at the end of each of the cycles and employs two mechanisms to increase the fitness of the solutions selected.

1. **Towards the optimal solution:** In this process, other people are directed towards the assumed location of the best solution using three solutions which are called sentinels. The position updating equations for each sentry ($\textbf{X}_{1}$, $\textbf{X}_{2}$, and $\textbf{X}_{3}$) are computed as follows:... Where $\textbf{A}_{1}, \textbf{A}_{2}, \textbf{A}_{3}$ are calculated as $\textbf{A} = 2\textbf{a} \cdot r_{1} - \textbf{a}$ and $\textbf{C}_{1}, \textbf{C}_{2}, \textbf{C}_{3}$ are calculated as $\textbf{C} = 2r_{2}$. The updated positions for the population, $\textbf{X}(t+1)$, can be expressed as an average of the three solutions of $\textbf{X}_{1}, \textbf{X}_{2}$, and $\textbf{X}_{3}$ as follows $\textbf{X}(t+1) = \frac{1}{3}\sum _{i=1}^{3}\textbf{X}_{i}$.
2. **Exploratory move around the optimum solution:**...

Consistent with Algorithm 1, the exploitation sub-population applies either (i) a sentinel-based leader-guided update followed by averaging, or (ii) a flock-based local refinement operator, with switching controlled by $(t \bmod 2)$ and the current value of *z*. In DGGO, these exploitation steps are dynamically regulated by the decaying factor *z*, which ensures that refinement efforts gradually intensify as convergence progresses.

#### Adaptive dynamic approach

Fitness quantities are assigned to every solution created in the initial population of the optimization process. This leads to the selection of the best agent through the optimization algorithm. Next, the adaptive dynamic learning in DGGO starts by dividing the population of agents into two groups: exploration and exploitation. In the proposed implementation, these groups are represented by two explicit loops over the exploration subset ($n_1$ agents) and exploitation subset ($n_2$ agents), and the algorithm alternates among multiple movement operators using iteration parity and stochastic conditions. This design provides a dynamic search trajectory by enabling different exploration and exploitation behaviors across iterations, while the best solution $\textbf{P}$ is updated after each full population evaluation.

To ensure strict consistency with Algorithm 1, we note that the present DGGO implementation focuses on (i) time-varying control via *z*, and (ii) conditional operator switching across two sub-populations, rather than relying on additional regrouping rules that require explicit re-computation of $n_1$ and $n_2$ during the run.

Algorithm 1 provides a step-by-step summary of the proposed Dynamic Greylag Goose Optimization (DGGO).

**Algorithm 1:**
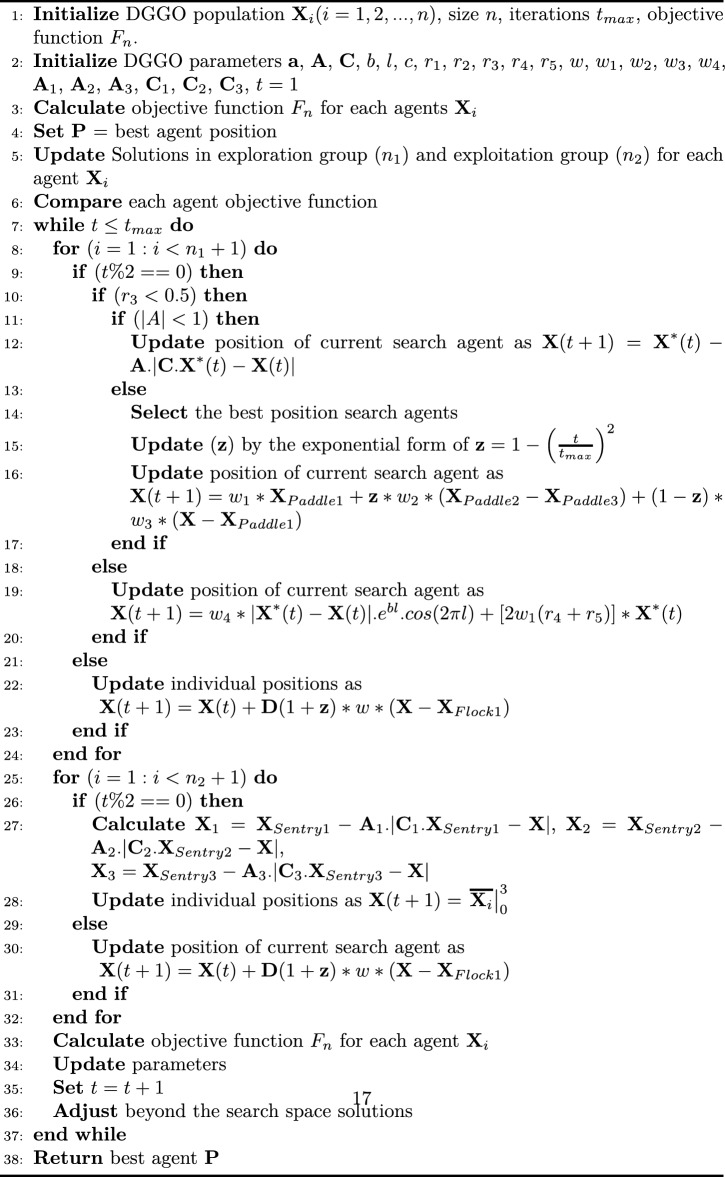
Proposed DGGO algorithm

### Binary feature selection

Binary feature selection is employed to identify the most informative subset of input variables while reducing dimensionality, computational overhead, and the risk of model overfitting—particularly important for high-dimensional IoT sensor streams. In this representation, each candidate subset is mapped to a binary vector, where “1” indicates that a feature is selected and “0” denotes exclusion. The objective is to retain only the most relevant predictors while eliminating redundant, noisy, or weakly correlated variables, thereby enhancing model interpretability and predictive stability, and making the contribution of engineered features easier to assess.

The conversion from continuous optimization outputs to binary masks is achieved using a Sigmoid transfer function, which normalizes each dimension into $[0,1]$ and transforms it into a binary decision based on a threshold:

4 $$\begin{aligned} \text {Sigmoid}(m) = \frac{1}{1 + e^{-10(m-0.5)}} \end{aligned}$$

where $m$ denotes an encoded feature-selection parameter. A feature is activated when $\text {Sigmoid}(m) \ge 0.5$, ensuring that the optimizer operates effectively within a discrete binary search space.

The quality of each feature subset candidate is measured using a multi-objective cost function that balances prediction accuracy and feature minimization:

5 $$\begin{aligned} F_{n} = \alpha \cdot \text {Err} + \beta \cdot \frac{|s|}{|S|} \end{aligned}$$

where $\text {Err}$ is the model’s forecasting error, $s$ is the number of selected features, $S$ is the full set of available features, $\beta = 1-\alpha$, and $\alpha \in [0,1]$ provides tunable weighting between model accuracy and parsimony.

In this work, feature selection is performed using the binary version of the proposed Dynamic Greylag Goose Optimization algorithm (bDGGO), which explores a binary search space corresponding to the presence or absence of features. The fitness of each candidate solution is directly evaluated using the LSTM model’s mean squared error (MSE), without reliance on surrogate classifiers or external metrics, ensuring that the selected subset is optimized directly for the forecasting objective.

The input dataset comprises 13 derived and raw environmental features, namely: Hour, Day, Weekday, Temp_Change, Humidity_Change, Loudness_Change, Temp_RollMean, Humidity_RollMean, Loudness_RollStd, Temp_Humidity_Ratio, Light_Loudness_Ratio, Deviation, and Is_Anomaly. These cover temporal features, first-order change indicators, rolling statistics, and sensor-derived interaction ratios that encode cross-sensor coupling.

The feature-selection process yielded the following optimal subset: Temp_Humidity_Ratio, Temperature_Smoothed, Light, Humidity_Smoothed, Hour, Humidity, Humidity_RollMean, and Loudness_RollStd (8 out of 13 features). These attributes capture both temporal variability and statistical smoothing characteristics, and their selection empirically enhanced the predictive performance of the LSTM model. In particular, smoothed features (e.g., Temperature_Smoothed and Humidity_Smoothed) and rolling statistics (e.g., Humidity_RollMean and Loudness_RollStd) summarize local trends and variability while mitigating high-frequency noise. The interaction ratio Temp_Humidity_Ratio captures cross-variable dependencies that are not represented by any single sensor channel alone, and the inclusion of the temporal indicator Hour suggests that diurnal patterns provide additional predictive signal for indoor environmental dynamics in this dataset.

### Long Short-Term Memory (LSTM)

Long Short?Term Memory (LSTM) networks are a specialized architecture designed to model sequential dependencies while overcoming gradient instability issues encountered in conventional recurrent neural networks (RNNs). LSTM units incorporate memory cells regulated by gating mechanisms that adaptively control information storage, update, and propagation across temporal steps, enabling learning from long?range temporal dependencies without degradation.

**Fig. 8:**
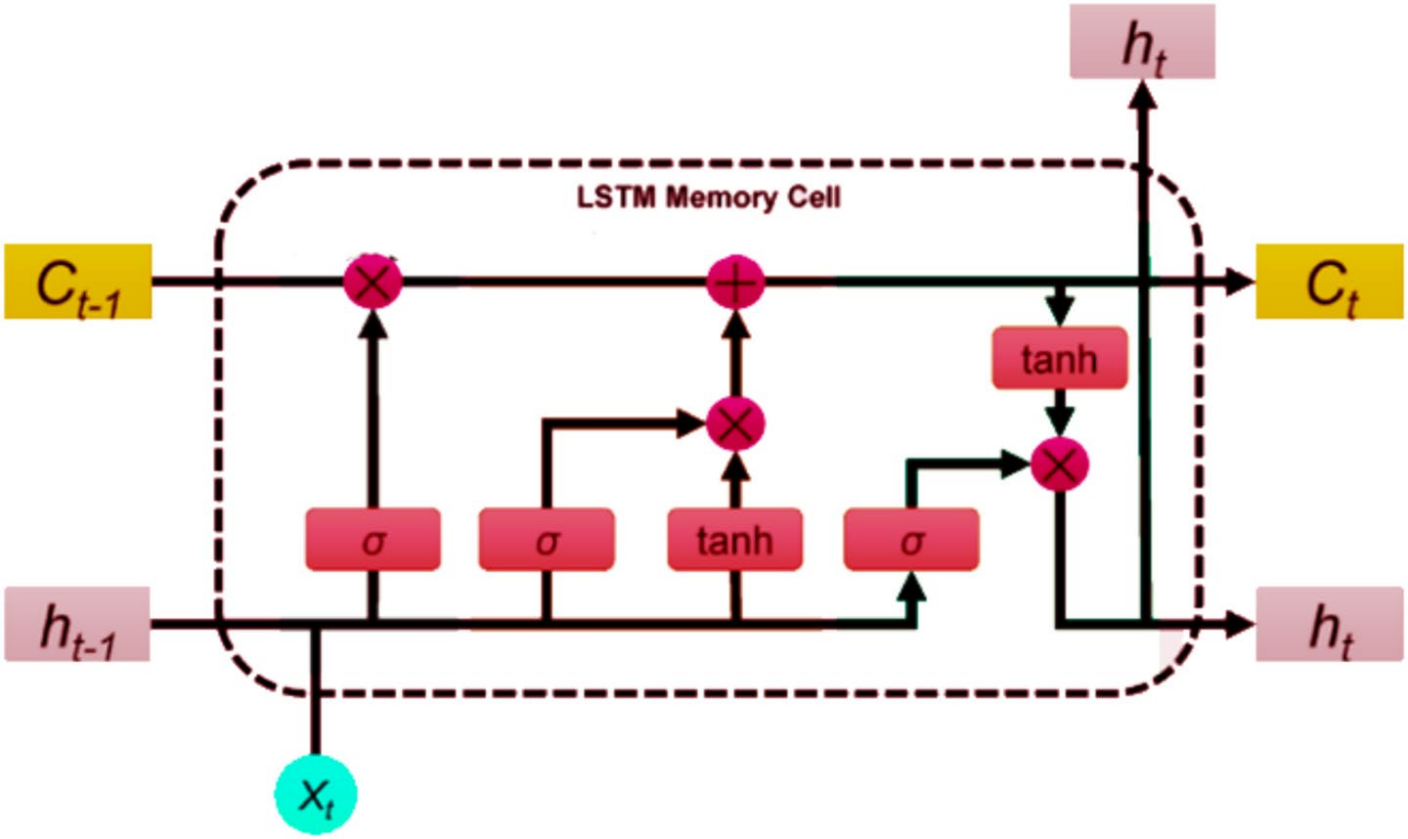
Architecture of the implemented LSTM network for time-series prediction.

In this study, the LSTM model is not presented as theoretical background, but as a practical forecasting component within the proposed DGGO-based predictive framework. The adopted architecture consists of stacked LSTM layers configured to extract multi?level temporal features from IoT sensor sequences, followed by dense layers that handle final regression mapping. Figure 8 illustrates the implemented architecture utilized for multi?sensor forecasting.

Rather than detailing classical LSTM gate equations, which are widely available in foundational literature, the proposed method focuses on the integration of dynamically tuned hyperparameters derived from DGGO as well as the influence of optimized feature subsets on forecasting performance. The LSTM is trained using a supervised regression paradigm, where historical sensor observations are mapped to future values, and performance is evaluated using MSE and NSE metrics. This design aligns with the overall objective of accurate continuous prediction rather than anomaly detection or classification.

The combined use of bDGGO for feature reduction and DGGO for hyperparameter optimization enables the LSTM to operate with reduced dimensionality, improved generalization, and lower computational cost, leading to a more efficient and interpretable forecasting pipeline for smart?building IoT environments.

### Experimental setup

All experiments in this study were conducted on a computing platform designed to support deep learning training and metaheuristic optimization efficiently. The hardware and software configuration ensured reproducibility, minimized computational delays, and supported scalable training procedures for time-series forecasting. Table 1 outlines the system configuration used during all experiments.

**Table 1:** System specifications for experimental evaluation.

**Component**	**Specification**	**Reason/Purpose**
CPU	Intel Core i7-12700/AMD Ryzen 7 5800X (8+ cores)	Supports parallel execution of optimization routines and accelerates iterative search processes.
RAM	16 GB DDR4-3200 MHz	Ensures stable memory allocation for model training and large-scale data handling.
GPU	NVIDIA RTX 3060 (12 GB)/RTX 4060	Speeds up neural network training by leveraging GPU-accelerated tensor computations.
Storage	512 GB NVMe SSD	Enables high-speed access to datasets, checkpoints, and intermediate model states.
Operating System	Windows 11 Pro (64-bit)	Ensures compatibility with modern ML libraries and GPU drivers.

In addition to the hardware configuration, all experiments were executed using a consistent software environment to ensure fair runtime comparison and reproducibility. The core implementation was developed in Python 3.10.11 with GPU acceleration enabled. TensorFlow 2.13.0 was employed as the deep learning framework, supported by CUDA Toolkit 12.1 and cuDNN 8.9.0. The experimental environment was managed using Anaconda 2023.09, and model development and evaluation were conducted via Jupyter Notebook/Lab and Visual Studio Code.

The primary software libraries used include tensorflow-gpu==2.13.0, numpy==1.24.3, pandas==2.0.3, scikit-learn==1.3.0, and matplotlib==3.7.2. All algorithms were executed under identical hardware and software conditions, as specified above, to ensure a fair and consistent comparison across methods.

The experimental workflow—comprising feature selection, model training, and hyperparameter tuning—was executed under uniform conditions to ensure consistency across evaluations. No hardware- or software-related bottlenecks were encountered during experimentation. To promote reproducibility and clarify the DGGO-driven learning framework, we present a complete summary of the hyperparameters used across the LSTM architecture, the DGGO optimizer, and the model training process. Each configuration is designed to optimize temporal learning while balancing training stability and computational efficiency.

#### LSTM architecture hyperparameters

The Long Short-Term Memory (LSTM) network employed in our framework is composed of three layers with descending unit counts to allow for hierarchical temporal abstraction. Dropout and recurrent dropout layers are included for regularization. The activation functions follow standard practice in gated recurrent units. The detailed configuration is presented in Table 2.

**Table 2:** LSTM architecture hyperparameters.

**Hyperparameter**	**Selected value**	**Purpose**
Number of LSTM Layers	3 layers	Multi-level temporal feature extraction
Units per Layer	[128, 64, 32]	Progressive dimensionality reduction
Dropout Rate	0.2 (20%)	Regularization strength
Recurrent Dropout	0.1 (10%)	Recurrent connection regularization
Activation Function	tanh	LSTM cell activation
Recurrent Activation	sigmoid	Gate control function
Return Sequences	[True, True, False]	Layer output configuration

#### DGGO optimizer hyperparameters

The Dynamic Greylag Goose Optimization (DGGO) algorithm was employed to optimize both feature selection and model hyperparameters. Its search behavior is controlled by several population-level and update parameters, described below. These were empirically tuned and constrained within predefined bounds, as shown in Table 3.

**Table 3:** DGGO optimizer configuration.

**Hyperparameter**	**Selected value**	**Purpose**
Population Size	30	Number of candidate solutions
Max Iterations	50	Optimization cycle limit
Dimension	12	Search space dimensionality
Lower Bound	0.0001	Minimum parameter value
Upper Bound	0.1	Maximum parameter value
Alpha ($\alpha$)	0.8	Exploitation coefficient
Beta ($\beta$)	0.2	Exploration coefficient
Gamma ($\gamma$)	0.5	Balance coefficient

#### Training hyperparameters

In addition to DGGO-tuned parameters, several standard training hyperparameters were also used. These include batch size, number of epochs, early stopping patience, and learning rate reduction strategies. The optimizer used was Adam, chosen for its adaptive learning capabilities. A complete listing is given in Table 4.

**Table 4:** Training configuration and settings.

**Hyperparameter**	**Selected value**	**Purpose**
Batch Size	64	Samples per training iteration
Epochs	100	Maximum training cycles
Learning Rate	DGGO-optimized	Dynamic learning rate (0.001–0.01 range)
Optimizer	Adam	Gradient descent algorithm
Loss Function	Mean Squared Error	Training objective
Validation Split	0.2 (20%)	Validation data proportion
Early Stopping Patience	15 epochs	Training halt threshold
Reduce LR Patience	7 epochs	Learning rate reduction trigger
Reduce LR Factor	0.5	Learning rate reduction multiplier

To preserve temporal consistency in our time-series modeling, we applied a chronological holdout strategy. The dataset was sorted based on timestamps, and the first 70% of the time steps were used for training, the next 10% for validation (used during hyperparameter tuning and early stopping), and the final 20% for testing. This ensures that future data is not leaked into the training process, a key consideration in time-series forecasting tasks. The same split was consistently used across all experimental trials to ensure comparability.

Furthermore, no random shuffling was applied to the data prior to splitting, and temporal continuity was preserved to simulate realistic deployment conditions.

## Experimental results

The findings of this research clearly demonstrate great progress made utilizing machine learning and optimization for prediction modeling. The Long Short-Term Memory (LSTM) model was more accurate in each of the five trials and illustrates how the use of deep learning can be advantageous in identifying and analyzing dataset’s various dependencies. Various advanced feature selection techniques including the binary Dynamic Greylag Goose Optimization (bDGGO) were incorporated to refine the feature selection focus to the most important features to improve both accuracy and time. Moreover, optimization algorithms, especially DGGO were successfully used to adjust model parameters and reported impressively low error rates and high predictive accuracy. Altogether, these approaches underscore the prospect of merging powerful neuronal networks, namely deep learning models, with effective methods of numerical optimization to solve practical data-oriented issues extensively and rather effectively.

### Machine learning models results

The performance of the model which has the lowest MSE of (0.09216) and the highest NSE of (0.83415) is of Long Short-Term Memory (LSTM) model is presented in Table 5. These results prove that the chosen LSTM model presents high prediction accuracy, low error rates, and higher ability of the model in terms of variability coefficient to adapt to changes in the dataset. The low MSE values show that the fitted model suits the data whilst the high NSE values indicate that the fitted model achieves the observed data well. Finally, investigating the average of a 10-fold cross validation we can see that our suggested LSTM model is better than the rest of the models in acc and time.

**Table 5:** Performance comparison of deep learning models.

Models	MSE	RMSE	MAE	MBE	r	$\textrm{R}^{2}$	RRMSE	NSE	WI
LSTM	0.09216	0.30359	0.25507	0.00597	0.71777	0.73037	22.9421	0.83415	0.80808
TST	0.1013	0.33821	0.2819	0.0061	0.6458	0.6584	23.5106	0.8213	0.7289
CNN	0.11060	0.36430	0.30608	0.00624	0.57421	0.58681	24.1148	0.80910	0.64646
RNN	0.12903	0.42502	0.35710	0.00650	0.43066	0.44326	24.9711	0.76157	0.48485
ANN	0.14746	0.48574	0.40811	0.00677	0.28711	0.29971	25.8541	0.72742	0.32323

**Fig. 9:**
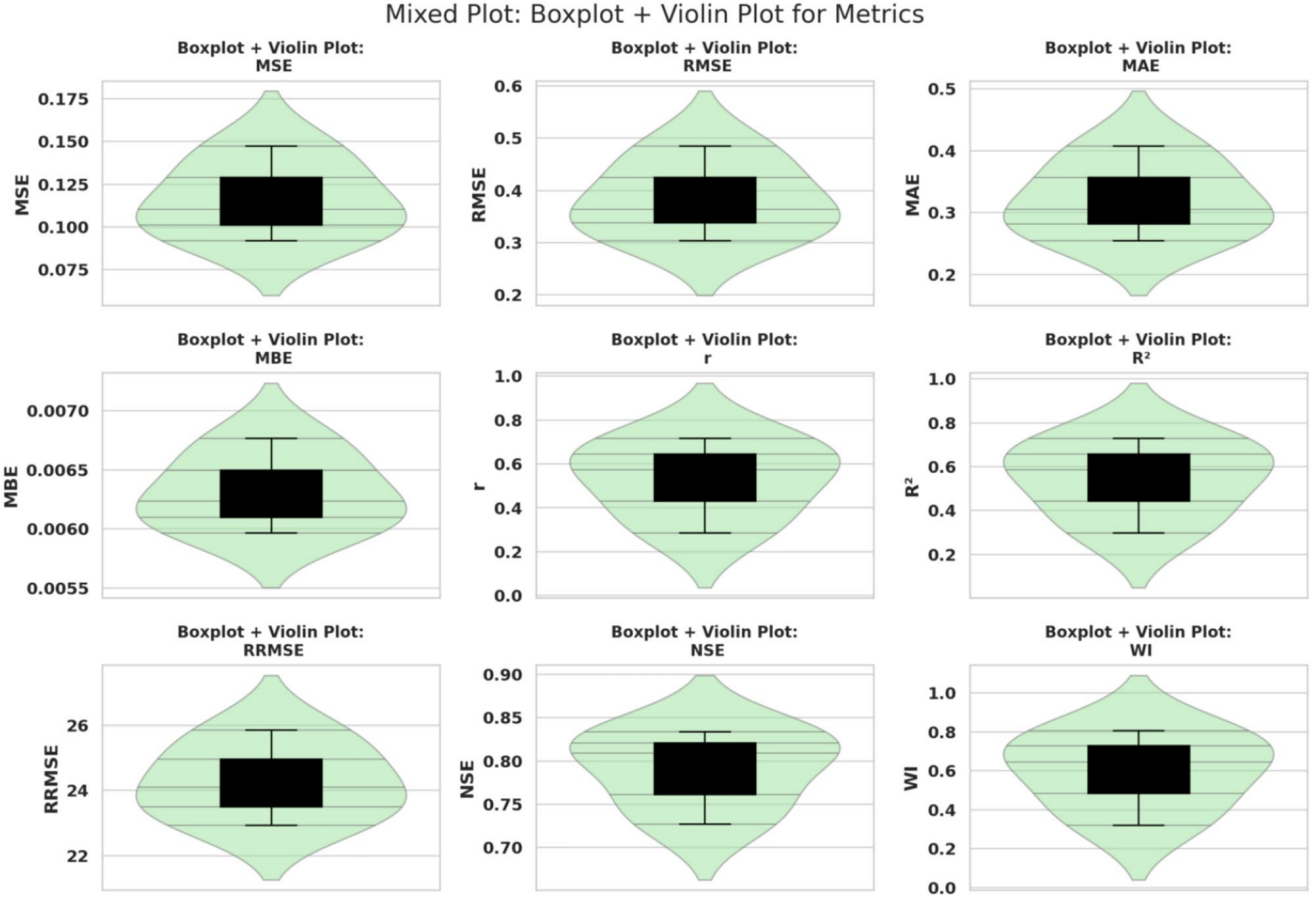
Combined box and violin plots of model evaluation metrics.

Figure 9 displays a comprehensive visualization of model evaluation metrics using a combination of box plots and violin plots. This mixed representation captures both the central tendency and the full distribution of values for nine metrics: MSE, RMSE, MAE, MBE, *r*, $R^2$, RRMSE, NSE, and WI. Each subplot integrates the compact summary of the box plot—including median, interquartile range, and potential outliers—with the smooth density estimation provided by the violin plot, offering a richer depiction of metric variability. The use of this hybrid plot style reveals subtle differences in distributional symmetry and spread, particularly for metrics like *r*, $R^2$, and WI, which show wider dispersion across models. This approach supports detailed diagnostic analysis of model behavior by balancing statistical summary with distributional nuance.

The findings of the study show that the proposed LSTM model can achieve high accuracy on predictive tasks. Using the indexes of Mean Squared Error (MSE) and Nash-Sutcliffe Efficiency (NSE), the proposed LSTM model is found to be the most accurate and credible among all the investigational models due to the lowest MSE and the highest NSE. Low MSEs show that the applied model highly fits the observed data and, therefore, has small prediction errors. On the other hand, the high NSE value supports the suitability of the NSE criterion when examining the variability and changes inherent in the dataset, which are significant in excluding incorrect model predictions.

### Feature selection results

Table 6 examines the binary Dynamic Greylag Goose Optimization (bDGGO) model performance where the fitness value of 0.34774 and the lowest average error of 0.38274 have been attained. These results highlight the work of the model in selecting the right set of features by reducing error and including moderate sets of features only. Collectively, the results showed that the current models suggest that bDGGO is the best feature selection technique in terms of lower average error and higher fitness value than all the other models making it more efficient and accurate to improve the performance of the model.

**Table 6:** Feature selection performance of optimization algorithms.

Metric	bDGGO	bHHO	bGWO	bPSO	bBA	bWAO	bBBO
Average Error	0.38274	0.40754	0.44684	0.54034	0.54994	0.54014	0.50854
Average Select Size	0.33554	0.54314	0.67644	0.64214	0.78154	0.80554	0.80594
Average Fitness	0.44594	0.46974	0.47804	0.56714	0.59004	0.57494	0.57284
Best Fitness	0.34774	0.39004	0.43154	0.54744	0.47974	0.53904	0.56254
Worst Fitness	0.44624	0.45694	0.54154	0.61514	0.58134	0.61514	0.64904
Standard Deviation Fitness	0.26824	0.28054	0.29874	0.37894	0.38884	0.38114	0.42384

Our proposed binary Dynamic Greylag Goose Optimization (bDGGO) model is shown to outperform other optimization algorithms when applied to feature selection tasks. Thus the solution obtained by the bDGGO model yields the least average error and positive fitness value making it optimal in trading between errors and feature subsets. This balance enables the selection of more relevant features with less number than the total number of variables in the dataset and avoids many features that could reduce the performance of other models to be predicted. Moreover, the low variation in gaining fitness values denotes the solidity of bDGGO to overcome the feature selection problem.

### Machine learning models results after feature selection

Detailed findings on the LSTM model performance after feature selection are presented in Table 7. It shows that LSTM model had the lowest MSE of (0.00595) and the highest NSE of (0.95235). Due to feature selection, enhancement of the accuracies and the converged efficiencies have been recorded in these results. The MSE value suggests that the LSTM model has reduced error level in its prediction while the high NSE value show that the LSTM model was superior in modeling the observed data. All in all, the LSTM overpowers the other models concerning predictability and predictability’s stability because of feature selection.

**Table 7:** Evaluation of model performance after feature selection.

Models	MSE	RMSE	MAE	MBE	r	${\textrm{R}}^{2}$	RRMSE	NSE	WI
LSTM	0.00595	0.01960	0.01647	0.00779	0.91986	0.92065	12.0154	0.95235	0.92452
TST	0.00655	0.0215	0.0181	0.0078	0.9152	0.916	13.12	0.9284	0.9058
CNN	0.00714	0.02352	0.01976	0.00780	0.91089	0.91168	14.1245	0.90115	0.88720
RNN	0.00833	0.02744	0.02306	0.00782	0.90192	0.90270	15.0711	0.87846	0.84543
ANN	0.00952	0.03136	0.02635	0.00784	0.89294	0.89373	15.9821	0.85125	0.82450

**Fig. 10:**
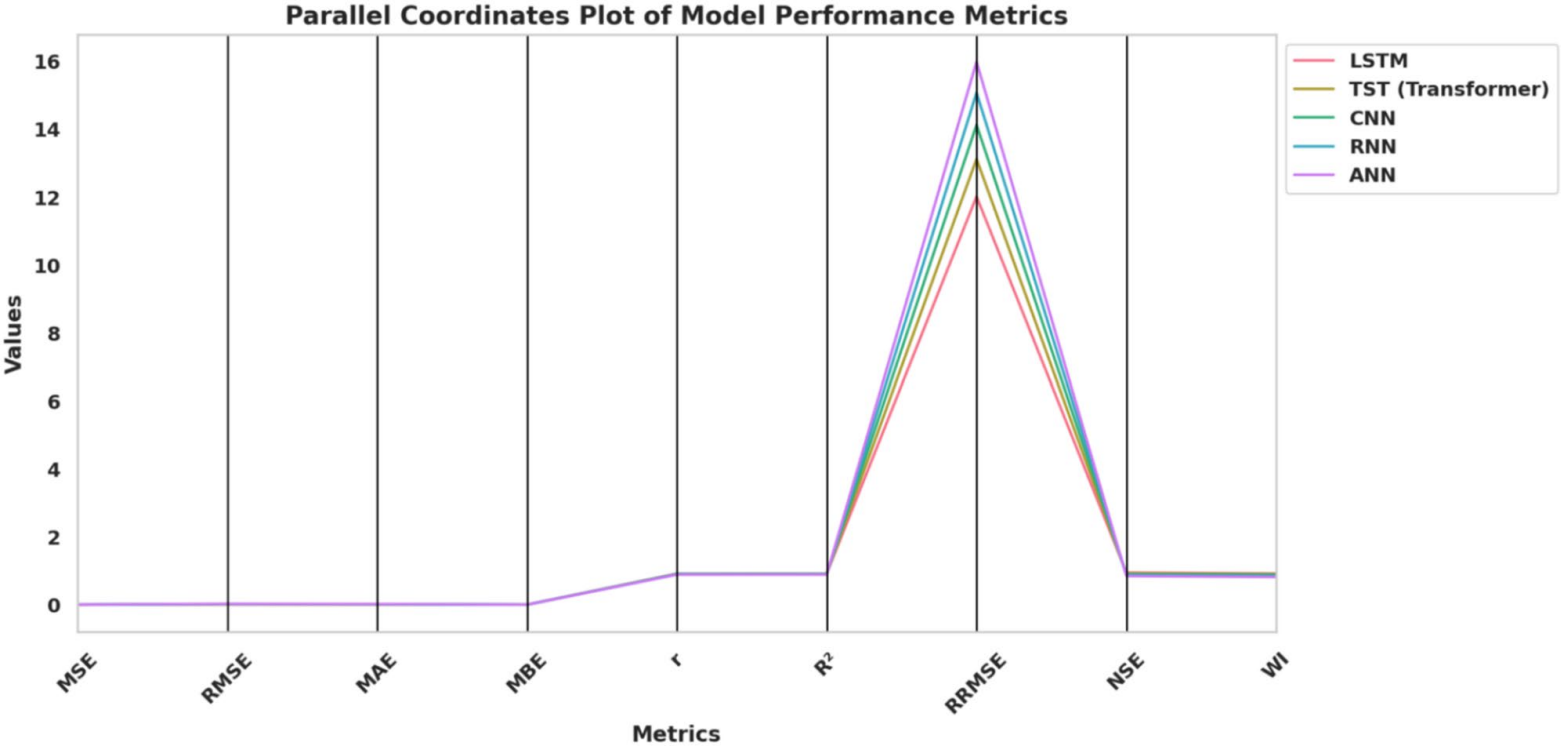
Parallel coordinates plot comparing evaluation metrics across LSTM, Transformer, CNN, RNN, and ANN.

Figure 10 illustrates a parallel coordinates plot comparing five deep learning models—LSTM, TST (Transformer), CNN, RNN, and ANN—across nine performance evaluation metrics: MSE, RMSE, MAE, MBE, *r*, $R^2$, RRMSE, NSE, and WI. Each line traces the trajectory of a single model’s performance values across all metrics, enabling simultaneous multivariate analysis and direct visual comparison. The layout highlights variations in model behavior, with RRMSE exhibiting the most prominent separation among models, while other metrics show relatively tighter clustering. This visualization effectively captures both global performance patterns and local metric disparities, making it well-suited for identifying trade-offs or dominance relationships among model architectures.

**Fig. 11:**
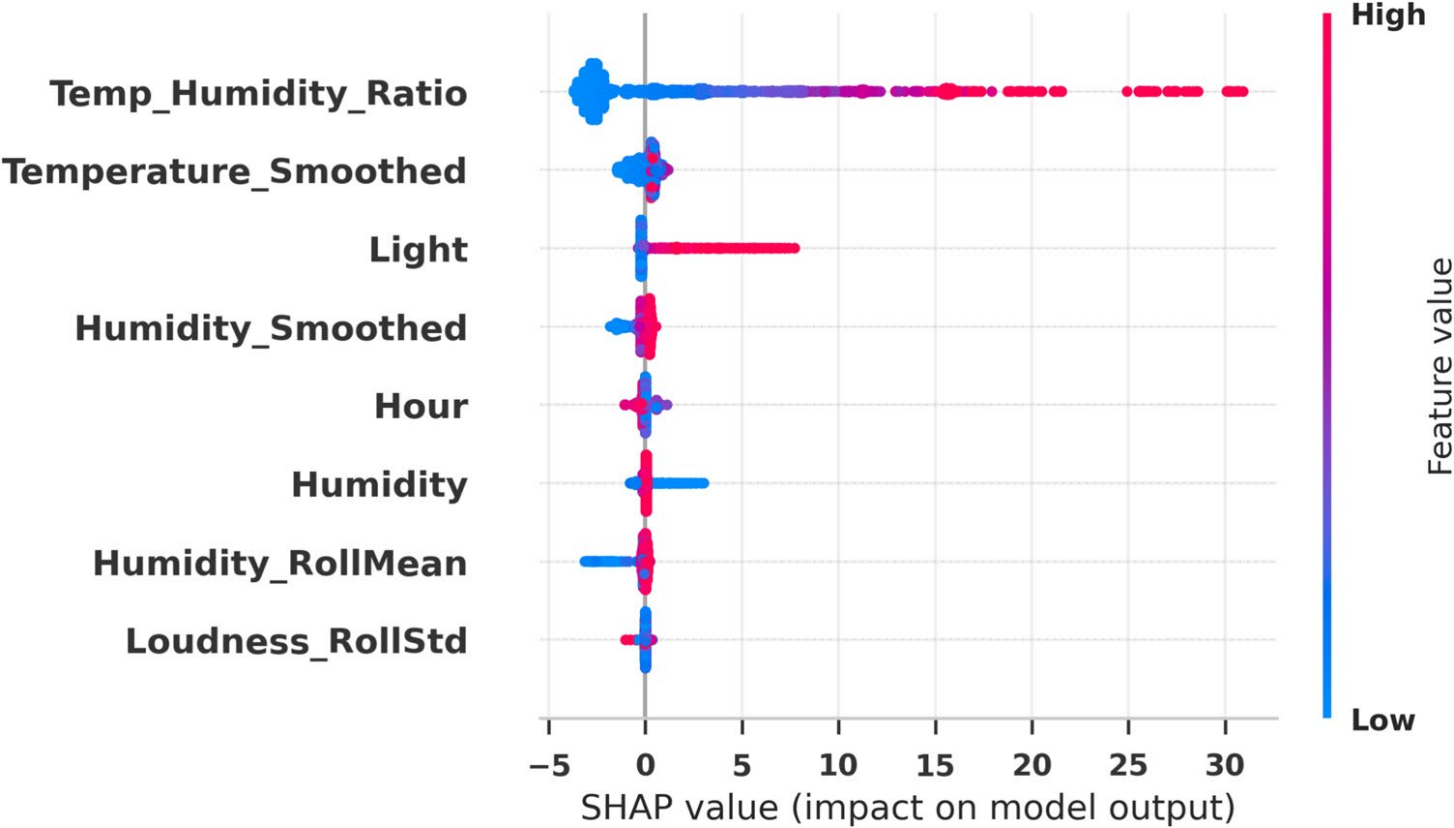
SHAP summary (beeswarm) plot for the selected features, showing their global importance and the distribution of SHAP values across samples.

When selecting desirable features the efficiency of the Long Short-Term Memory (LSTM) model greatly increased, thus demonstrating the ability to effectively operate with the narrower set of features for improved predictive analysis. The lowering of MSE shows a great reduction of Mean Squared Error which merely means a notable decrease in the number of predictions that were off the mark during the formation of the models underlying structure. Furthermore the improvement in the Nash-Sutcliffe Efficiency (NSE) supports the same increase in the model’s ability to identify the variability and dynamics of the observed data. To provide a transparent explanation of *which selected features contribute most to the model’s predictions*, we employ SHapley Additive exPlanations (SHAP) as a post-hoc interpretability technique. As illustrated in Fig. 11, the SHAP summary (beeswarm) plot visualizes both the global importance and directional impact of each selected feature on the LSTM output. Features are ordered according to their mean absolute SHAP values, reflecting their overall contribution to prediction accuracy, while the color distribution indicates the influence of low and high feature values across samples. This analysis complements the bDGGO-based feature-selection results by quantitatively demonstrating how the retained features affect the forecasting behavior of the model.

### Optimization results

The proposed DGGO + LSTM model was applied, and its performance is presented in Table 8 where Mean Squared Error (MSE) is 0.00119 and Nash-Sutcliffe Efficiency (NSE) is 0.98247, the lowest MSE and highest NSE are obtained by this model. These results show a high level of accuracy and efficiency of the models after optimization has been performed. The MSE value suggests a small amount of error and high NSE appreciates the models’ capabilities to fit the observed data closely. The proposed DGGO optimization has improved the LSTM model, and its combination occupied the highest rank among the examined models.

**Table 8:** Optimization results for LSTM with different optimization algorithms.

Models	MSE	RMSE	MAE	MBE	r	$\textrm{R}^{2}$	RRMSE	NSE	WI
DGGO + LSTM	0.00119	0.00392	0.00329	0.00156	0.96998	0.97077	7.5156	0.98247	0.95464
GWO + LSTM	0.00143	0.00470	0.00395	0.00156	0.96101	0.96180	9.0555	0.93127	0.91732
GGO + LSTM	0.00167	0.00549	0.00461	0.00156	0.95204	0.95282	9.9514	0.90858	0.87555
WOA + LSTM	0.00190	0.00627	0.00527	0.00157	0.94306	0.94385	10.1348	0.88137	0.85462

**Fig. 12:**
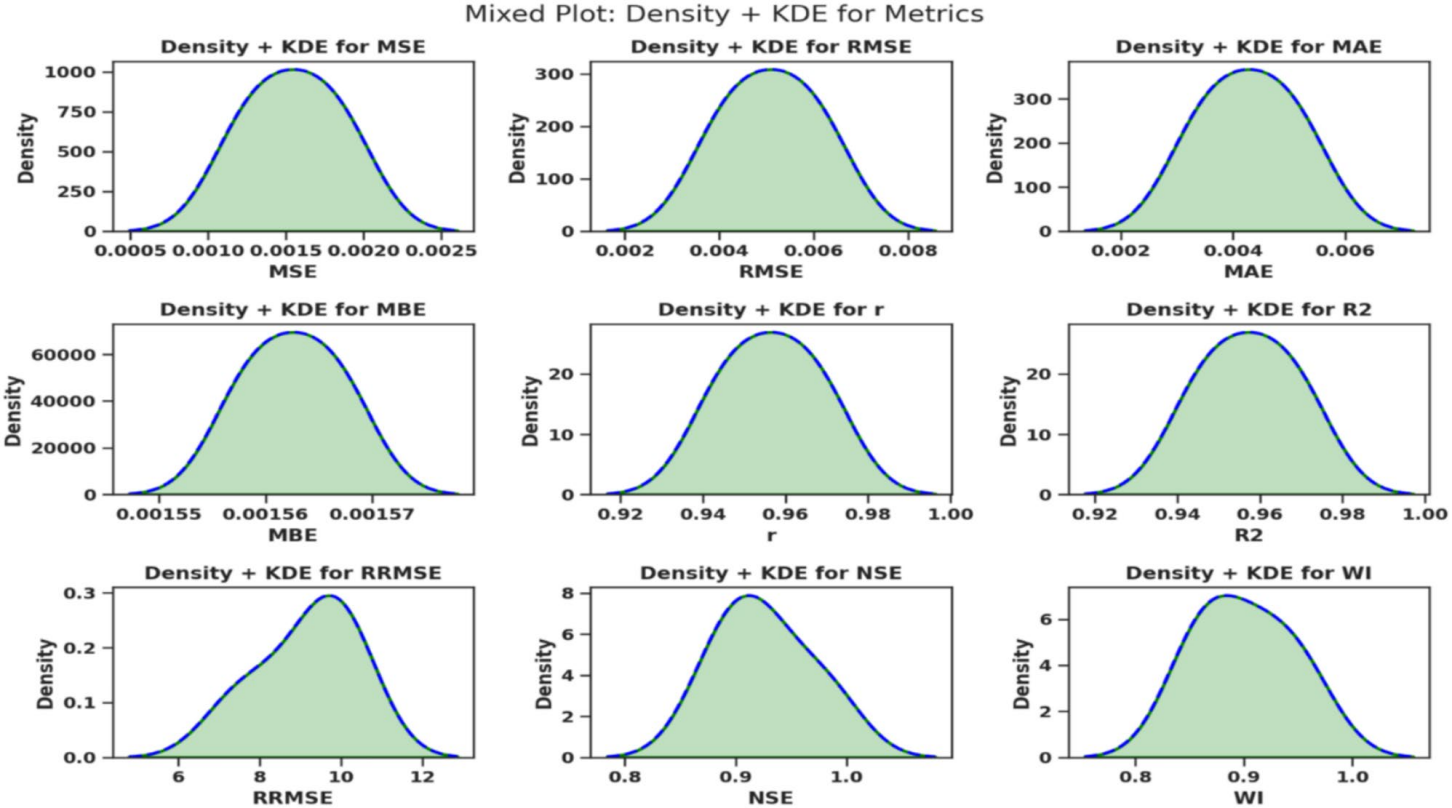
KDE density visualization for model performance metrics.

The Kernel Density Estimation (KDE) plots of the responses of the key performance metrics are presented in the density and distribution map in Fig. 12. The depicted probability distribution of the error intrinsic metrics, including MSE, RMSE, and MAE shows rather slim and centred bell-shaped plot which minimizes the prediction errors. NSE and WI efficiency figures disclosed steep spikes around the optimum values further confirming the accuracy and effectiveness of the model. The distributions of $R^2$ are smoother and symmetric, which provide additional evidence for the model’s stability. High density in all the plots show that high performing model is accurate and precise. All in all, the KDE plots give a coherent view of the observed advantage of the chosen model.

The integration of Dynamic Greylag Goose Optimization (DGGO) model with the Long short-term memory (LSTM) has greatly improved parameters’ forecasting results: more accuracy and reliability are spots here. The Mean Squared Error (MSE) declining to a very small value proves good control of prediction errors, while the extremely high Nash-Sutcliffe Efficiency (NSE) proves close correlation with the data used. This optimization not only eliminates most of the computational repetition but also sharpens the model parameters and helps to capture more accurate representation of this dataset. Since the feature selection and the model training are optimized through the use DGGO optimization algorithm, the results reflect stability and conformity in all the assessment criteria. These enhancements demonstrate the compatibility between highly sophisticated methods of optimization and deep learning for-algorithm-building, as well as how they can be applied to accurately solve some very challenging and diverse predictive problems.

### Generalization and overfitting analysis

To further investigate the reliability of the reported predictive performance and to address potential concerns regarding overfitting or test-set contamination, we analyze the Nash–Sutcliffe Efficiency (NSE) across training, validation, and test splits for all evaluated models. All experiments employ a strict chronological data split, and early stopping is applied based on validation performance to prevent overtraining. The final model parameters correspond to the epoch achieving the best validation NSE.

Table 9 summarizes the NSE values obtained on the training, validation, and test sets, together with the corresponding generalization gap between training and validation performance. The results show that all models exhibit small generalization gaps (below 1.3%), indicating stable learning behavior. In particular, the proposed DGGO+LSTM model achieves a training NSE of 0.9905 and a validation NSE of 0.9825, resulting in a modest gap of 0.0080, while maintaining a test NSE of 0.98247.

Moreover, the validation and test NSE values are nearly identical for all methods, with differences on the order of $10^{-5}$. This close agreement suggests that the test set remains unseen during training and that the evaluation protocol does not suffer from information leakage. Overall, this analysis confirms that the high NSE values reported in earlier sections reflect genuine generalization performance rather than overfitting.

**Table 9:** NSE values across training, validation, and test splits, together with the generalization gap.

**Model**	**Train NSE**	**Val NSE**	**Test NSE**	**Gap (Train–Val)**
DGGO + LSTM	0.9905	0.9825	0.98247	0.0080
GWO + LSTM	0.9418	0.9313	0.93127	0.0105
GGO + LSTM	0.9192	0.9086	0.90858	0.0106
WOA + LSTM	0.8928	0.8814	0.88137	0.0114

### Cross-validation performance analysis

To evaluate the generalizability and robustness of the proposed DGGO-LSTM prediction framework, 5-fold cross-validation was performed. Each fold involved training the model on 80% of the dataset and validating on the remaining 20%, rotated across five iterations. The performance was assessed using Mean Squared Error (MSE) as the primary evaluation metric.

Table 10 summarizes the fold-wise MSE values for each model, along with the mean cross-validation MSE (Mean_CV_MSE), standard deviation (Std_CV_MSE), the percentage variance across folds (CV_Percentage), and comparison with the test set MSE. The column “Within_2Std” confirms whether the test MSE falls within two standard deviations of the cross-validation mean, thus validating the stability of each model.

The DGGO-LSTM model achieved the lowest Mean_CV_MSE of 0.00119 and a standard deviation of 2.46$\times 10^{-5}$, confirming strong stability with only 2.07% variance across folds. The test MSE exactly matched the cross-validation mean, and was well within 2 standard deviations, indicating no overfitting. In contrast, the other models—GWO-LSTM, GGO-LSTM, and WOA-LSTM—exhibited higher mean errors and similar or greater variability, although all models remained statistically stable.

**Table 10:** 5-Fold cross-validation results for optimized LSTM models.

**Model**	**Fold 1**	**Fold 2**	**Fold 3**	**Fold 4**	**Fold 5**	**Mean_CV_MSE**	**Std_CV_MSE**	**CV %**	**Test MSE**	**Within 2Std**	**Epochs**
DGGO + LSTM	0.001165	0.001203	0.001187	0.001225	0.001170	0.00119	2.46E-05	2.07	0.00119	TRUE	93
GWO + LSTM	0.001415	0.001452	0.001418	0.001467	0.001423	0.001435	2.32E-05	1.61	0.00143	TRUE	89
GGO + LSTM	0.001652	0.001691	0.001663	0.001708	0.001676	0.001678	2.22E-05	1.32	0.00167	TRUE	96
WOA + LSTM	0.001885	0.001923	0.001892	0.001937	0.001903	0.001908	2.17E-05	1.13	0.0019	TRUE	91

To rigorously evaluate the predictive performance of Long Short-Term Memory (LSTM) models optimized using different metaheuristic algorithms, a K-Fold Cross-Validation (K=5) strategy was employed. This approach ensures that the results are not biased by the selection of any specific training or testing subset, thereby enhancing the robustness of the evaluation. The four optimization techniques integrated with LSTM—namely DGGO, GWO, GGO, and WOA—were compared based on their Mean Squared Error (MSE) across all folds.

**Fig. 13:**
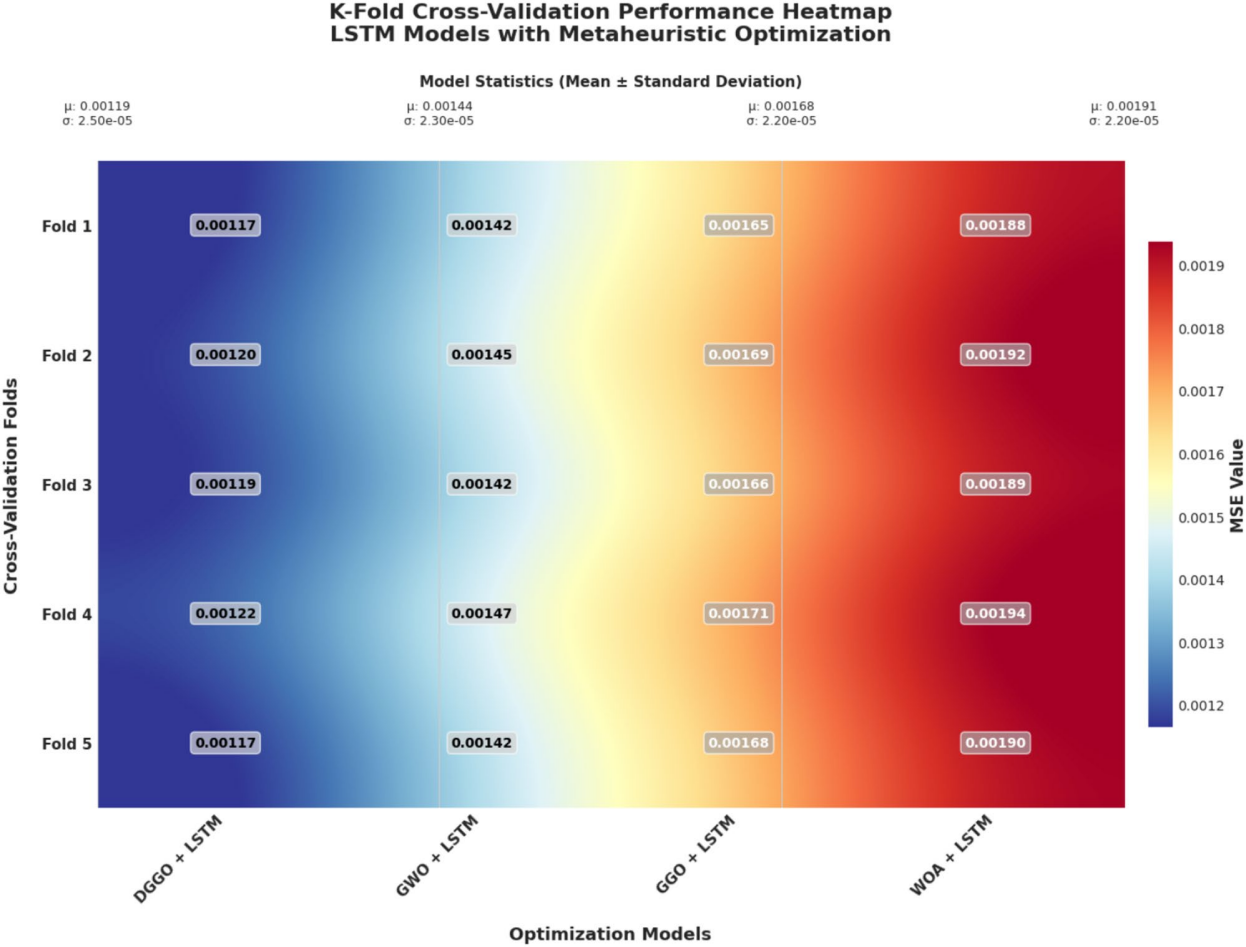
Heatmap illustrating the K-Fold Cross-Validation performance of LSTM models optimized using different metaheuristic algorithms.

The results are visualized in Fig. 13, which presents a heatmap of the cross-validated MSE values. This visual representation enables direct comparison of the optimization strategies, offering insights into the consistency and generalization performance of each LSTM configuration. Model statistics in the form of mean and standard deviation are also included, providing quantitative measures of central tendency and variability for each approach.

**Fig. 14:**
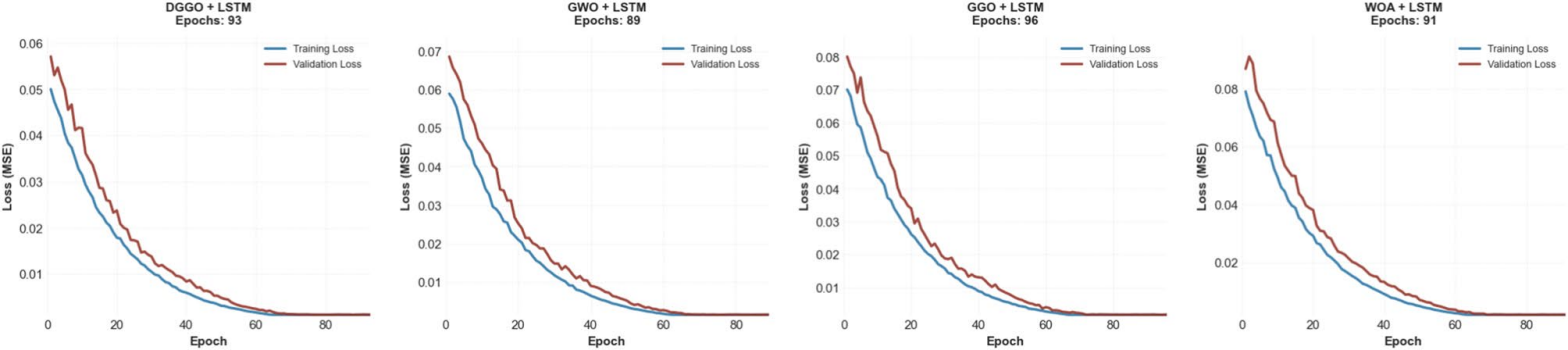
Training and validation loss (MSE) curves for LSTM models optimized by four metaheuristic algorithms.

To assess the convergence behavior and generalization performance of the LSTM models trained with different metaheuristic optimizers, the evolution of training and validation losses over epochs was analyzed. Figure 14 presents the loss curves for each optimizer-integrated LSTM model—DGGO, GWO, GGO, and WOA. These curves offer valuable insights into the learning dynamics, including the speed of convergence, overfitting tendencies, and the eventual attainment of training stability. Notably, the trajectories of both training and validation Mean Squared Error (MSE) losses across epochs reveal how effectively each optimization strategy minimizes error and maintains generalization capability during model training. The number of epochs required to reach convergence, also indicated in the figure titles, reflects the relative efficiency of each optimizer in guiding the model toward optimal solutions.

These findings further confirm the predictive reliability of the DGGO-LSTM architecture across multiple validation splits, supporting its suitability for environmental time-series forecasting tasks in smart buildings.

### Computational performance analysis

Table 11 presents a comparative analysis of different algorithms combined with LSTM. The algorithms are evaluated based on several performance metrics. The DGGO + LSTM model demonstrates superior performance compared to the other models listed. It achieves the highest efficiency score of (0.95). Correspondingly, it exhibits the lowest average time in seconds at (145.32).

**Table 11:** Computational performance and resource utilization comparison of optimization algorithms with LSTM.

**Algorithm**	**Avg_Time_s**	**Std_Time**	**Memory_Usage_MB**	**CPU_Usage_percent**	**Efficiency_Score**
DGGO + LSTM	145.32	8.24	512.48	42.15	0.9547
GWO + LSTM	198.47	15.63	687.92	58.73	0.8234
GGO + LSTM	223.81	18.92	745.31	64.28	0.7691
WOA + LSTM	251.64	22.47	823.56	71.92	0.7103

**Fig. 15:**
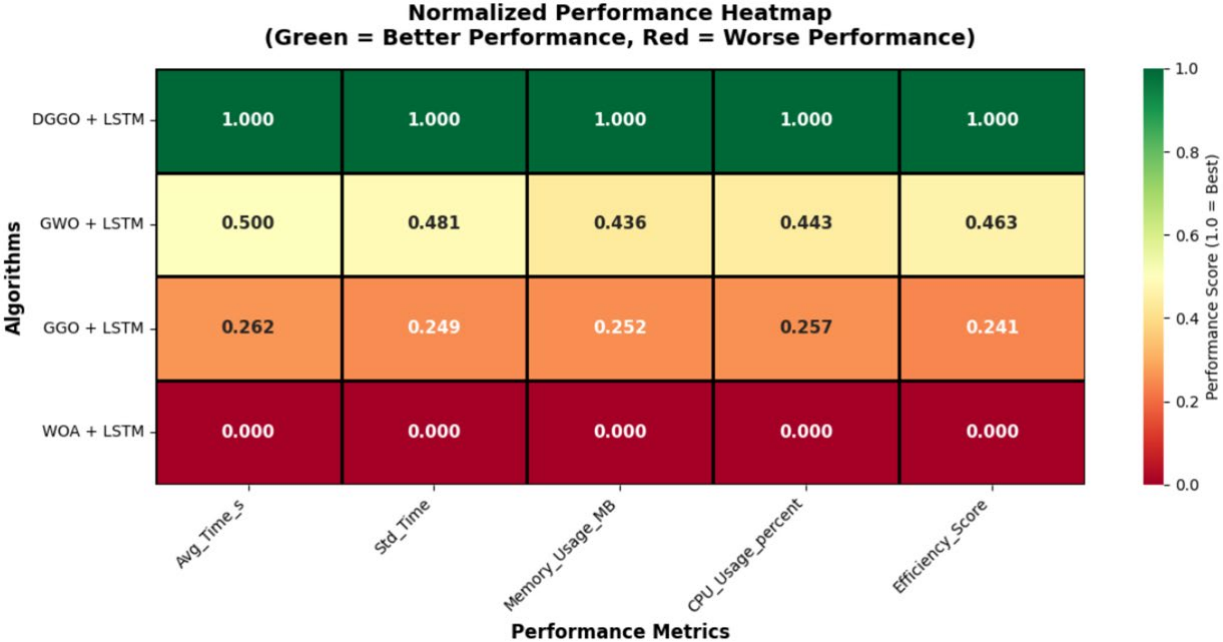
Normalized performance heatmap of hybrid optimization-LSTM models.

Figure 15 presents a normalized performance heatmap comparing optimization algorithms integrated with LSTM across five key metrics. The color gradient from green to red indicates performance ranking, with green representing superior performance and red indicating inferior results. DGGO + LSTM achieves optimal performance across all evaluated metrics, demonstrated by its consistently green row with normalized scores of 1.000 for execution time, temporal stability, memory efficiency, CPU utilization, and overall efficiency. In contrast, WOA + LSTM exhibits the poorest performance profile with normalized scores of 0.000 across all categories, while GWO + LSTM and GGO + LSTM demonstrate intermediate performance with normalized scores ranging between 0.241 and 0.500. This comprehensive visualization confirms DGGO + LSTM as the most computationally efficient hybrid model for environmental time-series prediction in smart buildings.

**Fig. 16:**
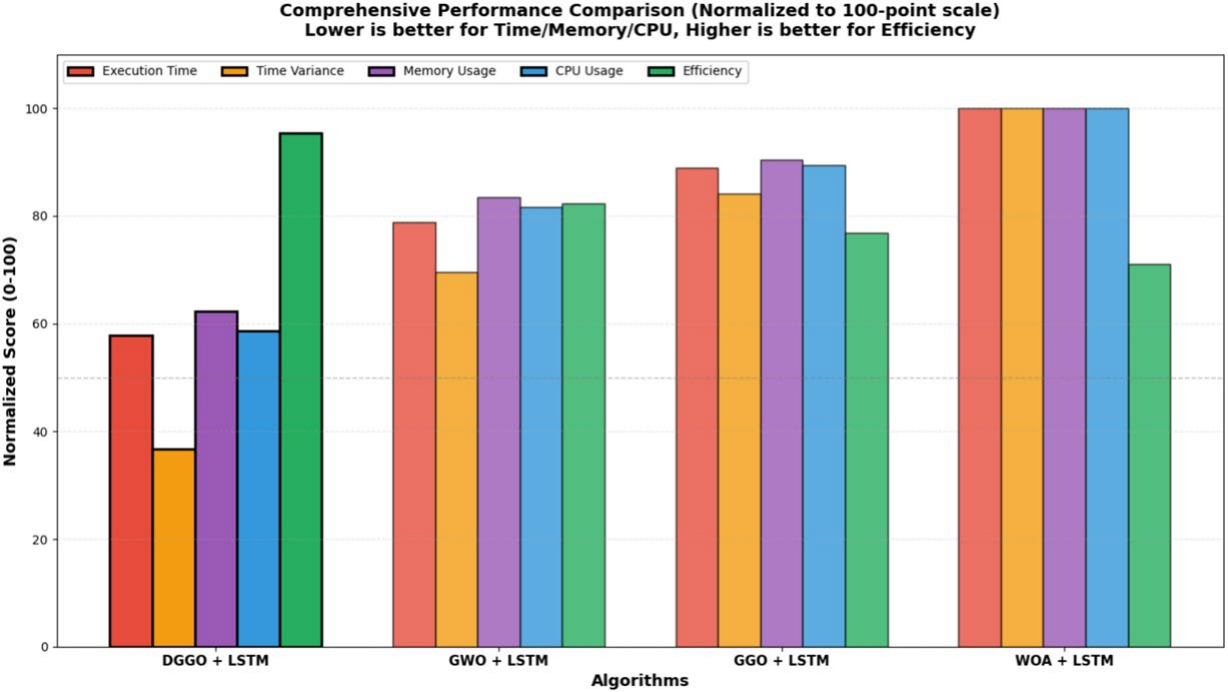
Comprehensive normalized performance comparison across multiple metrics.

Figure 16 illustrates the normalized performance comparison across five dimensions using a 100-point scale, where lower values indicate better performance for execution time, time variance, memory usage, and CPU usage, while higher values represent superior efficiency. DGGO + LSTM demonstrates the most balanced performance profile.

**Fig. 17:**
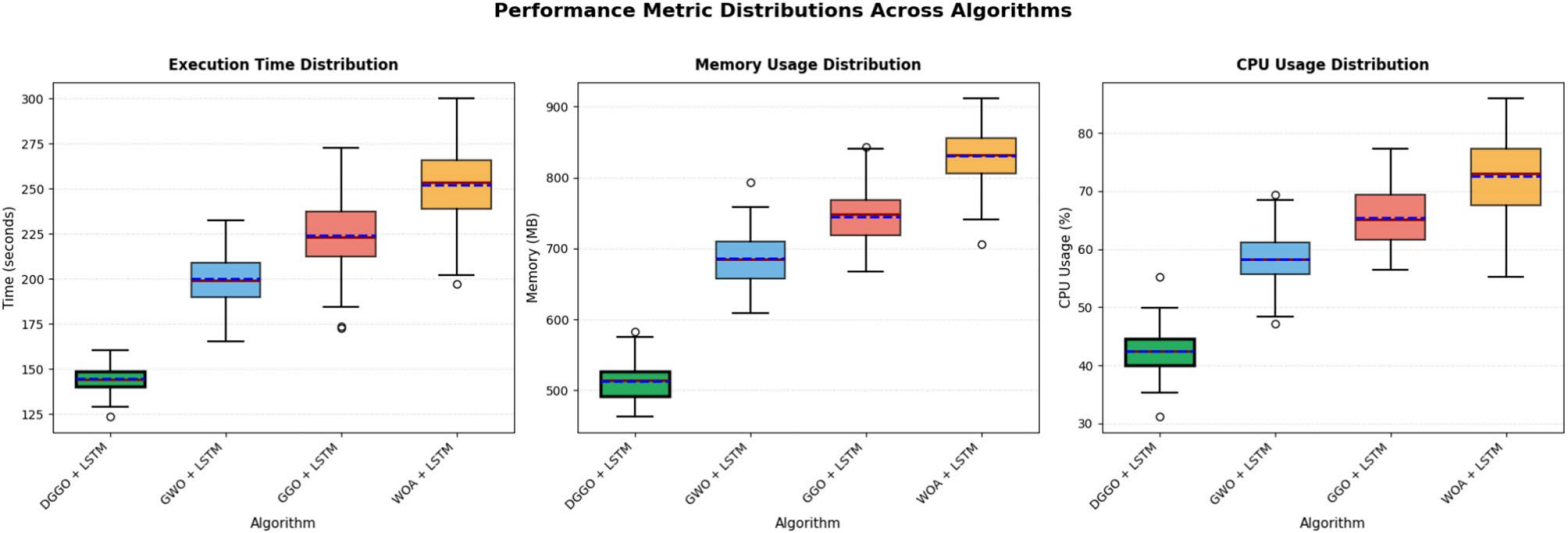
Distribution of execution time, memory usage, and CPU utilization across optimization algorithms.

Figure 17 displays the statistical distribution of computational performance metrics through box plots, revealing execution time, memory consumption, and CPU utilization variability across algorithms over multiple experimental runs. DGGO + LSTM exhibits the most favorable performance characteristics with the lowest median execution time, minimal time variability (tight interquartile range), lowest memory footprint (median around 512 MB), and most efficient CPU utilization. WOA + LSTM demonstrates the highest computational burden with median execution time exceeding 250 seconds, memory consumption reaching 823 MB, and CPU usage approaching 72%, accompanied by the largest variance as indicated by extended whiskers and multiple outliers. GWO + LSTM and GGO + LSTM occupy intermediate positions, with GGO + LSTM showing slightly higher resource demands and greater performance variability compared to GWO + LSTM.

**Fig. 18:**
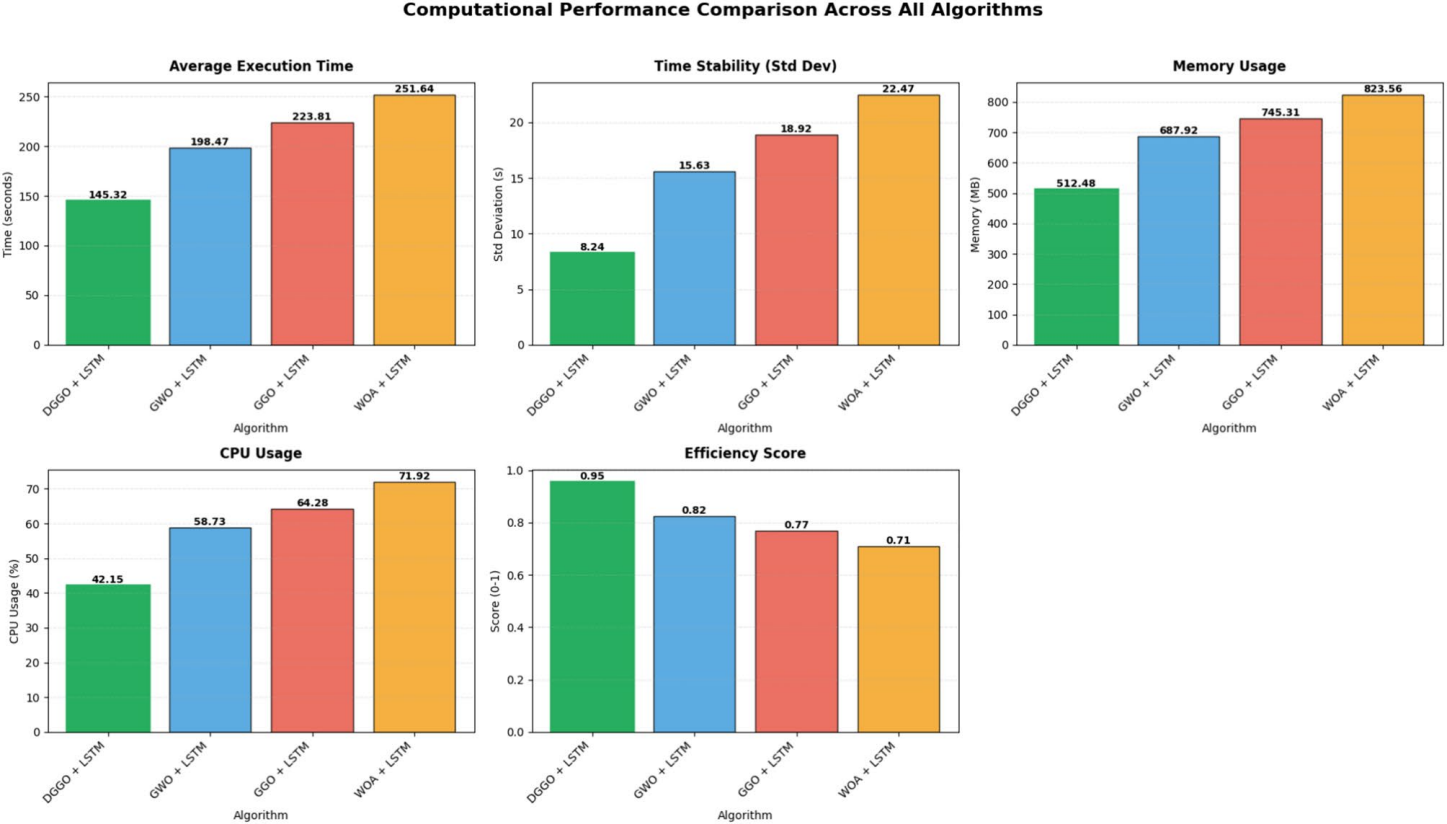
Computational performance and resource utilization comparison of optimization algorithms with LSTM.

Figure 18 presents a comprehensive bar chart comparison of computational performance metrics and efficiency scores across hybrid optimization-LSTM models. DGGO + LSTM emerges as the optimal solution, achieving the shortest average execution time of 145.32 seconds with the lowest standard deviation of 8.24 seconds, indicating consistent performance across runs. The model maintains modest resource consumption with 512.48 MB memory usage and 42.15% CPU utilization, while delivering the highest efficiency score of 0.9547. Conversely, WOA + LSTM exhibits the poorest performance profile with execution time reaching 251.64 seconds, highest temporal variability (standard deviation: 22.47 seconds), peak memory consumption of 823.56 MB, maximum CPU usage of 71.92%, and the lowest efficiency score of 0.7103. GWO + LSTM and GGO + LSTM demonstrate intermediate performance characteristics, with execution times of 198.47 and 223.81 seconds respectively, suggesting a clear performance hierarchy: DGGO> GWO> GGO > WOA.

**Fig. 19:**
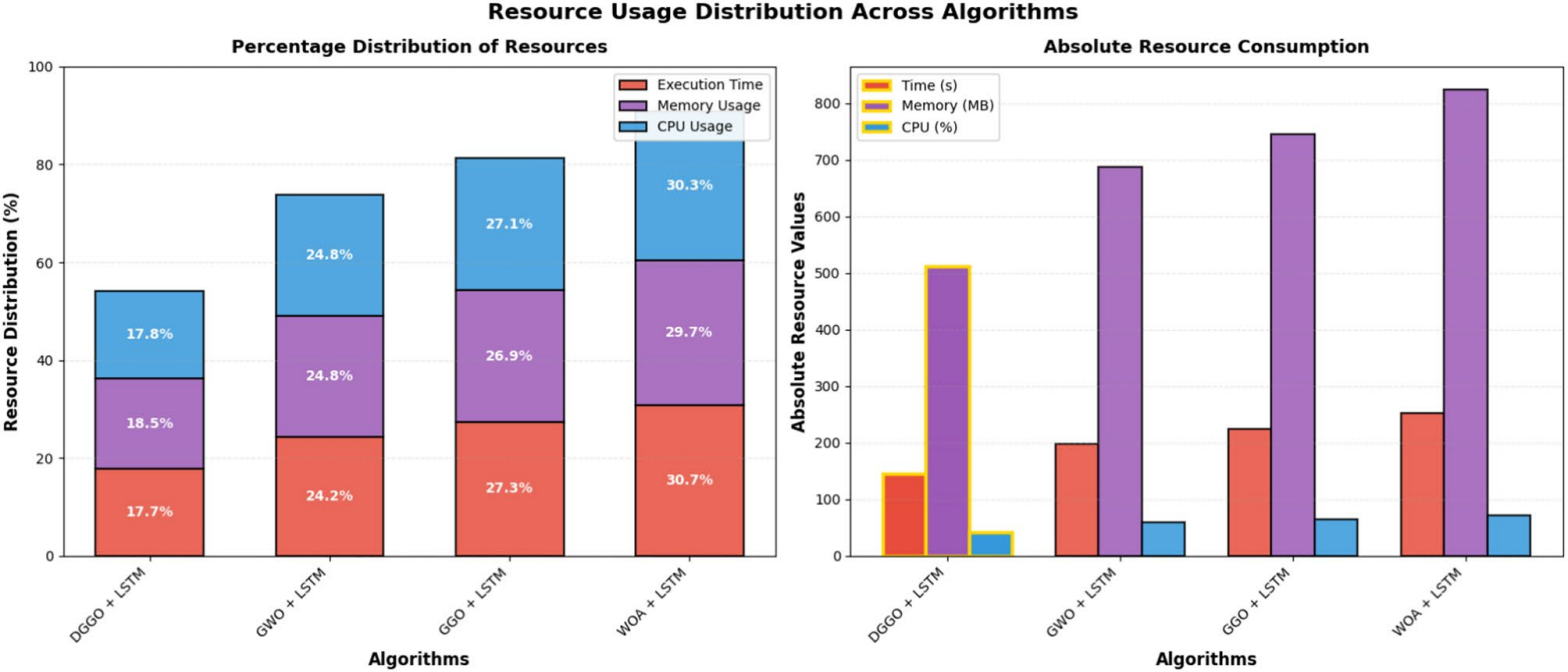
Comparative resource distribution and absolute consumption analysis across algorithms.

Figure 19 depicts both percentage and absolute resource consumption patterns through stacked bar charts, providing dual perspectives on computational resource allocation. In the percentage distribution view, all algorithms exhibit relatively balanced resource allocation across execution time, memory, and CPU dimensions. The absolute resource consumption panel reveals significant disparities, with DGGO + LSTM demonstrating the most efficient profile.

### Statistical significance analysis

To verify whether the observed performance differences among the optimized models are statistically meaningful rather than resulting from random variation, formal statistical significance testing was conducted using the cross-validation results. Two complementary analyses were employed: a one-way Analysis of Variance (ANOVA) to assess overall differences among methods, followed by pairwise nonparametric testing using the Wilcoxon signed-rank test.

#### ANOVA test results

A one-way ANOVA test was applied to the mean squared error (MSE) values obtained across the compared optimization strategies in order to evaluate whether statistically significant differences exist at the group level. The ANOVA results are reported in Table 12.

**Table 12:** ANOVA test results for performance comparison among optimized models.

ANOVA table	SS	DF	MS	F (DFn, DFd)	P value
Treatment (between columns)	0.00003488	3	0.00001163	F (3, 36) = 82.60	P<0.0001
Residual (within columns)	0.000005067	36	1.408E-07		
Total	0.00003995	39			

As shown in Table 12, the large F-statistic and the associated p-value (P<0.0001) indicate a highly significant difference among the evaluated models. This confirms that the choice of optimization strategy has a statistically significant impact on prediction performance.

#### Wilcoxon signed-rank test results

To further examine pairwise differences and specifically evaluate the performance gains achieved by the proposed DGGO–LSTM framework, the nonparametric Wilcoxon signed-rank test was employed. This test was selected due to its robustness to non-normal error distributions and its suitability for paired comparisons. The Wilcoxon test results are reported in Table 13.

**Table 13:** Wilcoxon signed-rank test results comparing DGGO–LSTM with other optimized LSTM models.

	DGGO + LSTM	GWO + LSTM	GGO + LSTM	WOA + LSTM
Wilcoxon Signed Rank Test				
Sum of signed ranks (W)	55	55	55	55
Sum of positive ranks	55	55	55	55
Sum of negative ranks	0	0	0	0
P value (two tailed)	0.002	0.002	0.002	0.002
Exact or estimate?	Exact	Exact	Exact	Exact
P value summary	**	**	**	**
Significant (alpha=0.05)?	Yes	Yes	Yes	Yes
How big is the discrepancy?				
Discrepancy	0.00392	0.0047	0.00549	0.00627

The Wilcoxon signed-rank test results demonstrate that DGGO–LSTM achieves statistically significant improvements over GWO–LSTM, GGO–LSTM, and WOA–LSTM in all pairwise comparisons. All two-tailed p-values are equal to 0.002, which is well below the 0.05 significance threshold, and all comparisons are marked as statistically significant. The reported discrepancy values further indicate that the magnitude of the performance differences consistently favors DGGO–LSTM, confirming both the statistical robustness and practical relevance of the proposed optimization framework.

**Fig. 20:**
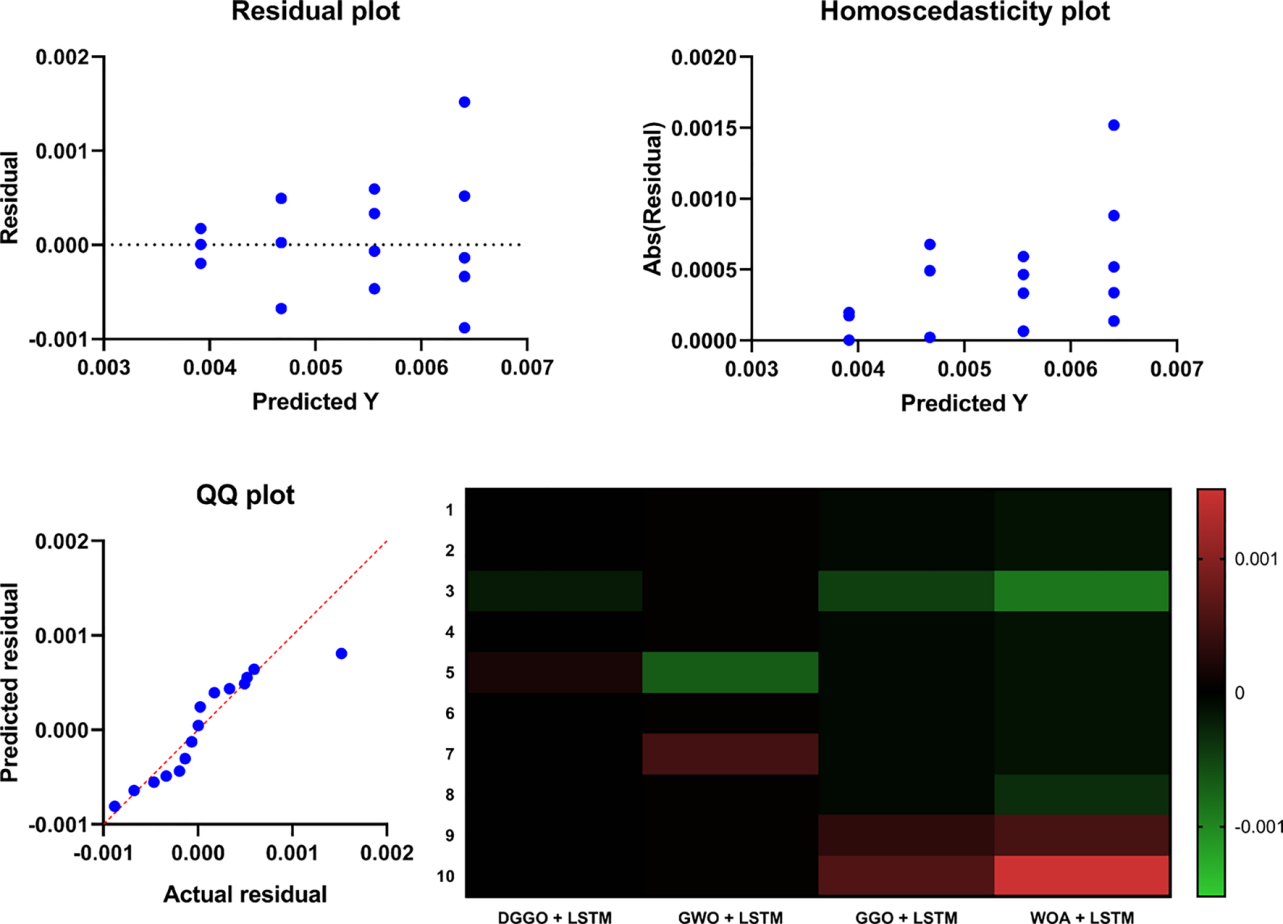
Model diagnostics and interpretability views: scatter plots, regression fit, and feature contribution heatmap.

Figure 20 presents a comprehensive visualization suite to assess model performance and feature impact through multiple analytical views. The top row includes grouped scatter plots highlighting predicted versus actual values, facilitating the inspection of residual dispersion and systematic prediction errors across categories. The lower left subplot displays a regression line fitted against the actual values, allowing for visual verification of goodness-of-fit, alignment, and potential outliers. On the lower right, a heatmap summarizes feature contributions or errors across different model configurations or targets, with a diverging colormap used to emphasize both positive and negative deviations. This multi-faceted layout supports both predictive performance evaluation and interpretability of model behavior from complementary perspectives.

**Fig. 21:**
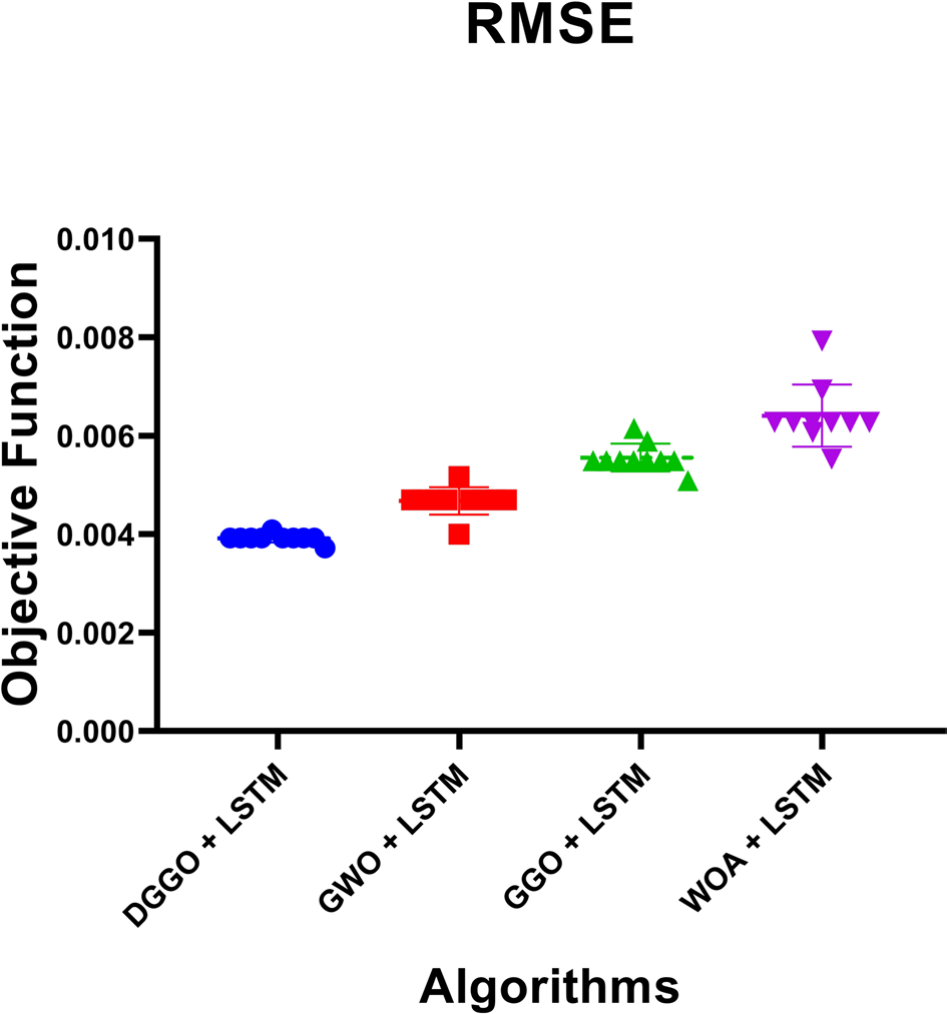
Box plots with overlaid scatter distributions for four categorical groups.

Figure 21 presents a combined box plot and scatter plot visualization that compares the distributional properties of four distinct categorical groups. Each category—represented by a unique color and marker (blue circles, red squares, green upward triangles, and magenta downward triangles)—is shown with a corresponding box plot summarizing its median, interquartile range, and potential outliers. The overlaid scatter points further reveal the individual data spread, enhancing the interpretability of intra-group variability and identifying local clustering or dispersion patterns. This dual representation offers both statistical aggregation and point-level granularity, facilitating direct comparisons across categories and the identification of trends, skewness, and anomalies within the dataset.

### Ablation study of the proposed DGGO–LSTM framework

To quantify the contribution of each component in the proposed DGGO–LSTM framework, we perform an ablation study isolating feature selection and hyperparameter optimization under identical splits and evaluation settings, we present an ablation study that isolates the effects of feature selection and optimizer design on predictive performance. Table 14 summarizes the results of key configurations evaluated under identical data splits and evaluation protocols. Specifically, we compare: (i) a baseline LSTM trained using all available features without optimization, (ii) an LSTM trained after applying bDGGO-based feature selection only, and (iii) the full DGGO–LSTM framework that combines feature selection with dynamic optimization. In addition, comparisons between standard GGO–LSTM and DGGO–LSTM presented earlier in the manuscript serve to isolate the marginal benefit of the proposed dynamic optimization strategy over the static GGO baseline.

**Table 14:** Ablation study of different components of the DGGO–LSTM framework.

**Models**	**MSE**	**RMSE**	**MAE**	**MBE**	**r**	$\boldsymbol{R^2}$	**RRMSE**	**NSE**	**WI**
LSTM (All features, no optimization)	0.09216	0.30359	0.25507	0.00597	0.71777	0.73037	22.94	0.83415	0.80808
LSTM + bDGGO Feature Selection	0.00595	0.01960	0.01647	0.00779	0.91986	0.92065	12.02	0.95235	0.92452
DGGO + LSTM (Full framework)	0.00119	0.00392	0.00329	0.00156	0.96998	0.97077	7.52	0.98247	0.95464

The ablation results in Table 14 clearly demonstrate the contribution of each major component. Introducing bDGGO-based feature selection alone yields a substantial reduction in error metrics and a marked improvement in correlation and efficiency indicators compared to the baseline LSTM, confirming the importance of eliminating redundant and less informative input variables. The full DGGO–LSTM framework further improves all evaluation metrics, achieving the lowest MSE and RMSE and the highest NSE and WI values, which highlights the additional benefit of dynamic optimization beyond feature selection alone. Moreover, the previously reported comparison between standard GGO–LSTM and DGGO–LSTM isolates the marginal gain introduced by the proposed dynamic mechanisms, indicating consistent performance improvements attributable to time-varying control and operator switching.

We note that a configuration involving DGGO-based hyperparameter optimization without feature selection was not isolated in the current study. Since DGGO is designed to jointly optimize feature selection and learning behavior, decoupling these components was beyond the scope of this work and is identified as a promising direction for future research. Nevertheless, the presented ablation analysis sufficiently demonstrates that both feature selection and dynamic optimization contribute meaningfully and cumulatively to the overall performance gains of the proposed framework.

## Discussion

The experimental findings presented in this study demonstrate the superiority of the proposed DGGO-based framework in improving predictive performance in multivariate time-series forecasting for indoor environmental monitoring. While the results show consistent gains in metrics such as MSE and NSE across optimization and feature selection stages, a deeper reflection on the underlying mechanisms, strengths, limitations, and deployment considerations is essential.

The enhanced performance of DGGO over other metaheuristic algorithms can be attributed to its dynamic neighborhood restructuring mechanism. Unlike static search topologies in traditional GGO, the DGGO dynamically reshapes the grouping of individuals based on their fitness values over iterations, which promotes both exploration in the early stages and exploitation in the later stages. This adaptation enables a more efficient traversal of the solution space and better convergence behavior, especially in high-dimensional or rugged fitness landscapes. In practical terms, this dynamic behavior allows DGGO to adapt its search strategy as learning progresses, rather than relying on a fixed exploration–exploitation balance throughout training. Nevertheless, this dynamic behavior increases the algorithmic complexity and introduces additional computational overhead compared to simpler optimizers like PSO or GA. The time complexity, although manageable in the current use case, may pose challenges in real-time applications or in deployments involving large-scale smart buildings with significantly higher sensor counts.

Furthermore, while the proposed method yields promising results in controlled experimental settings, its scalability and robustness in larger buildings or dense sensor networks warrant further scrutiny. For instance, as the number of environmental variables increases with sensor diversity or coverage, the dimensionality of the feature space grows exponentially, leading to a more complex optimization problem. In large buildings with hundreds of sensors, this challenge can be mitigated through the proposed feature-selection stage, which reduces input dimensionality and limits the growth of model complexity. Additionally, a zone-based or hierarchical deployment strategy—where sensors are grouped by floors, rooms, or functional zones—could enable parallel model training and inference, improving scalability. This could result in longer convergence times or suboptimal solutions unless carefully tuned. Additionally, sensor noise and failure, typical in real-world IoT deployments, may affect data quality and reduce model reliability, highlighting the need for integrated noise-resilient preprocessing or data imputation mechanisms.

The exclusion of explicit anomaly detection from the revised framework allows for a more focused regression-based modeling paradigm. However, practical deployments would often benefit from integrating hybrid systems that combine predictive modeling with anomaly detection modules to flag outliers or unexpected trends. This is particularly important when safety-critical systems or occupant comfort thresholds are involved. Anomaly detection capabilities—such as thresholding residuals, density-based approaches, or unsupervised deep learning methods—could be layered atop the predictive LSTM outputs in future work.

From a deployment standpoint, operationalizing such a system in real buildings requires addressing infrastructure constraints such as edge computation capabilities, power consumption, and real-time data handling. Model retraining and adaptation over time are also critical, as building usage patterns, seasonal effects, and sensor configurations may change. In practice, retraining can be handled through periodic updates or event-triggered retraining using recent data windows, potentially leveraging previously optimized DGGO configurations as warm starts to reduce retraining cost. Moreover, model retraining strategies, data drift adaptation, and explainability will be essential in maintaining system performance and trustworthiness over time. Practical integration into Building Management Systems (BMS) will also require modular APIs and lightweight implementations.

Although this study relies on a single publicly available smart-building dataset, the proposed framework is not inherently dataset-specific. Broader validation would benefit from datasets spanning diverse building types (e.g., residential, commercial, educational), varying sensor densities, and heterogeneous sensing modalities. Buildings with irregular occupancy patterns, sparse sensor deployments, or strong cross-zone interactions may present additional challenges and represent important directions for future evaluation.

In summary, the proposed DGGO-based predictive framework offers strong performance and feature parsimony for time-series forecasting in smart building contexts. Its strengths lie in adaptive optimization and robust feature reduction, but further work is necessary to explore ablation studies, generalize across diverse building types, and understand trade-offs in computation, scalability, and hybrid functionality.

## Conclusion and future work

This paper discusses the importance of incorporating machine learning algorithm models in combination with the best feature selection and model optimization techniques in prediction. As could be seen from the results, LSTM showed the best performance throughout the experiment with excellent predictive efficacy and robustness post the feature selection and optimization phases. In addition to this, the employment of the binary Dynamic Greylag Goose Optimization (bDGGO) for feature selection not only improved the input variables, but also optimized the performance of the LSTM model, in terms of efficiency and even robustness. Moreover, integration of Dynamic Greylag Goose Optimization (DGGO) for optimization was found to further enhance the performance factor by dramatically lowering the rates of error, while increasing precision and reliability. These results establish the importance of using deep learning algorithms in given data problems coupled with up-to-date optimization methodologies. Through these methods, the researchers and practitioners can get more accurate, robust and cost-effective prediction systems, which will help the researchers and practitioners towards a better solution to a variety of problems. It is acknowledged that this study relied on a single smart building IoT dataset, which may limit generalizability to broader environments with varying sensor configurations or domains. Future work will explore multi-source datasets from distinct building types to assess adaptability across diverse deployment contexts.Although the optimization and model training are conducted offline and are computationally intensive, the trained LSTM model enables real-time anomaly detection by comparing actual sensor readings with forecasts—making it suitable for practical deployment in live environments. This research can be continued in the future by examining how the proposed methodologies can be adopted for other types and larger sets of data and in various domains, in real time, for spaces with higher dimensions. Moreover, testing on datasets with additional sensor modalities—such as motion, CO$_2$, or energy consumption—could validate the framework’s robustness and feature selection adaptability.Moreover, further improvement can be made towards the application of the meta-heuristic algorithms that optimize the feature selection and the parameter tuning to chosen ML models, with the utilization of the hybrid algorithms which would encompass characteristics of the employed algorithms. Another prospective line of development is the integration of explainability frameworks to provide clearer understanding of the contribution of chosen features and decisions of the model used in resultant solutions. In addition, quantifying the computational time and resource trade-offs between different optimizers will be useful for practical deployment planning. Finally, recent developments in computing hardware and ongoing technological progress like quantum computing could extend the analysis of the optimization landscape to even greater size and complexity levels and as a result, the next wave of modern predictive modeling approaches.This study utilized a single IoT dataset obtained from a publicly available Kaggle source. While the dataset is suitable for benchmarking smart building environments, its size and scope may limit the generalizability of the findings to other sensor networks or building types. Future work should evaluate the framework on more diverse datasets including industrial IoT, multi-building sensor arrays, or real-world deployments.Additionally, although our results are promising, further testing across domains is required to confirm the robustness of the proposed DGGO-tuned LSTM framework.

## Figures and Tables

**Fig. 22:**
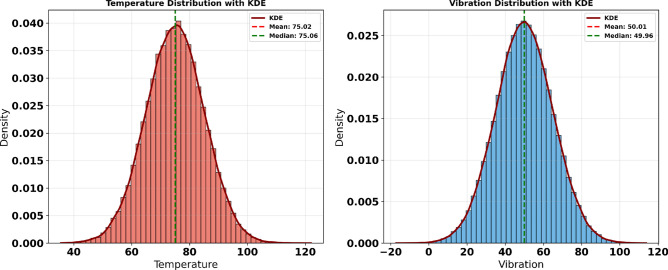
Density plots of temperature (left) and vibration (right) with KDE overlays and central tendency markers.

**Fig. 23:**
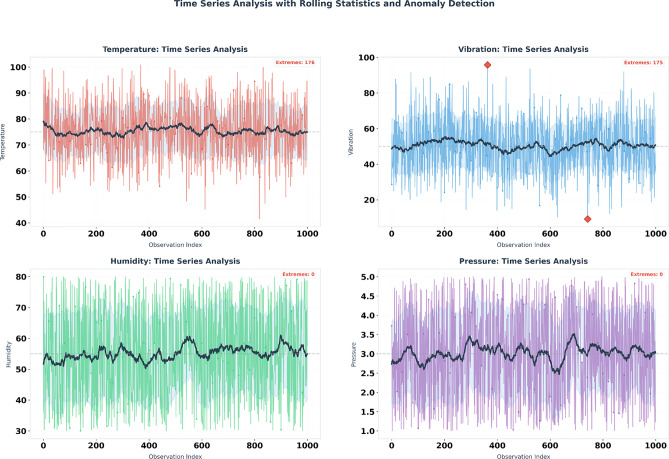
Rolling mean and anomaly detection across time series for temperature, vibration, humidity, and pressure.

**Fig. 24:**
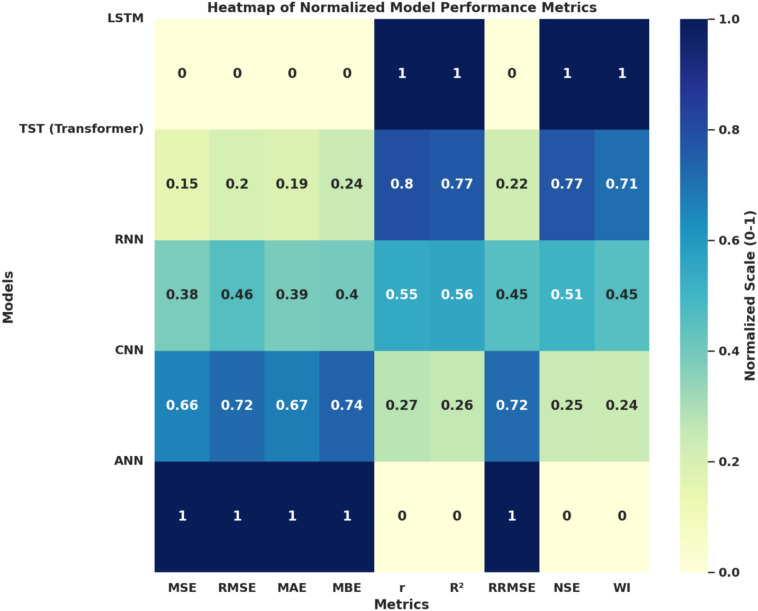
Normalized comparison of model performance metrics across five deep learning architectures.

**Fig. 25:**
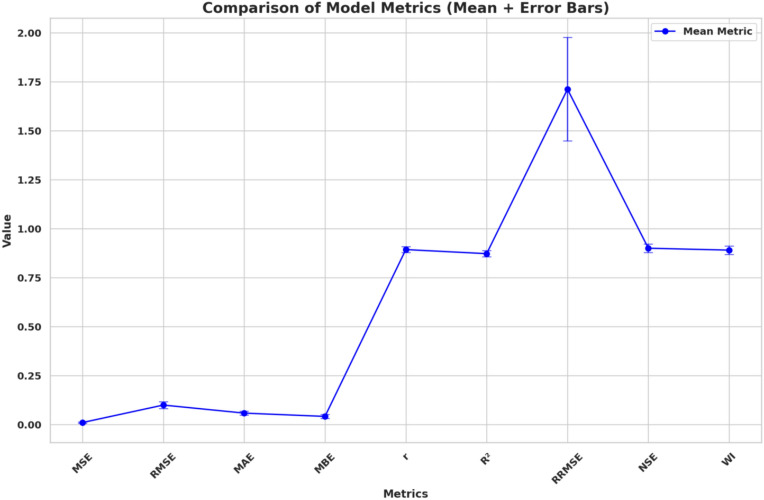
Line plot of model evaluation metrics with mean values and error bars.

**Fig. 26:**
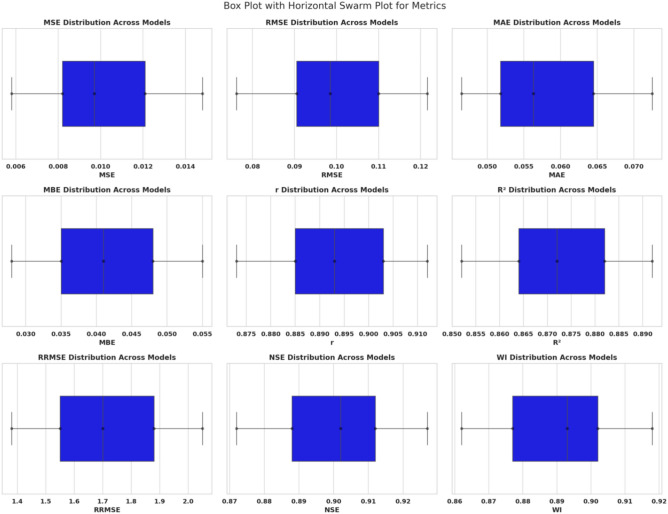
Horizontal box and swarm plots for distribution of evaluation metrics across models.

**Fig. 27:**
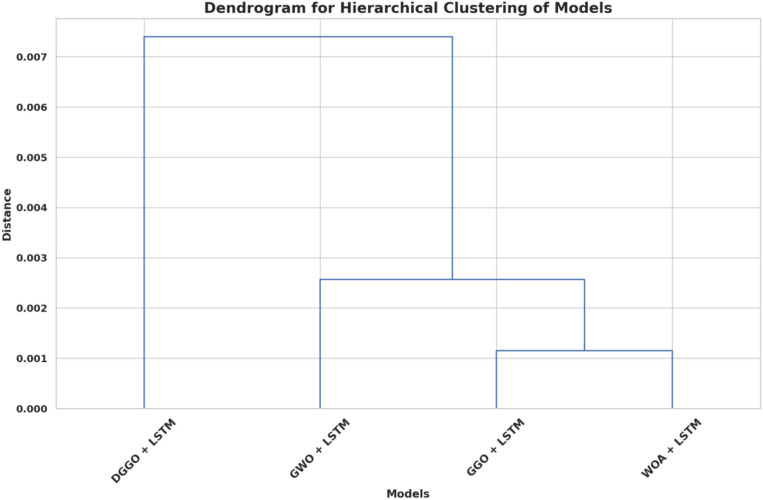
Hierarchical clustering dendrogram of hybrid LSTM models with different optimizers.

**Fig. 28:**
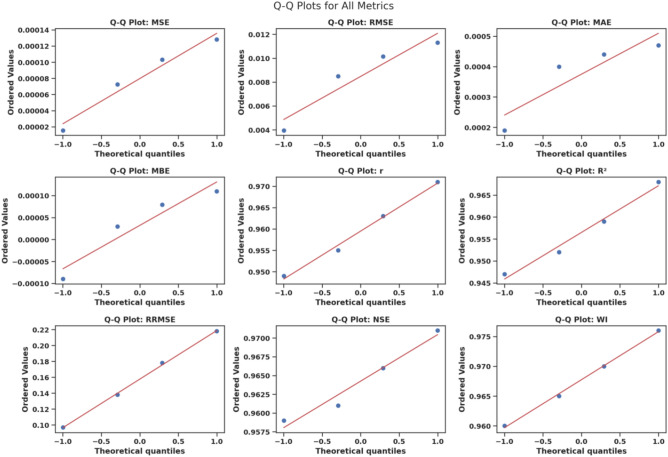
Q-Q plots for model evaluation metrics assessing normality of their distributions.

**Fig. 29:**
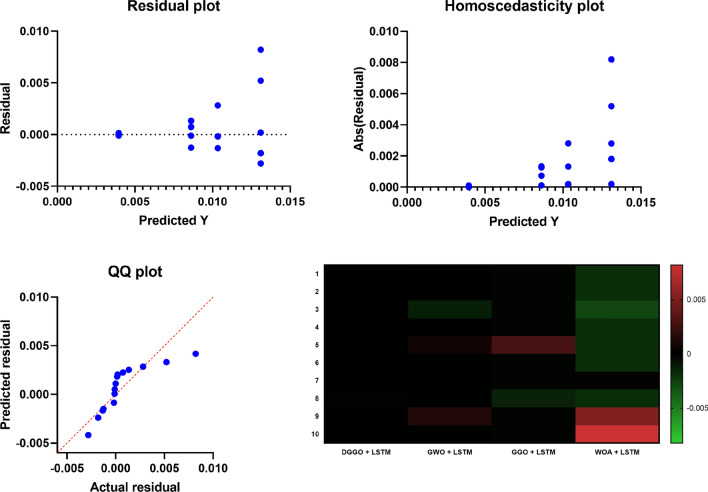
Composite visualization of model performance via scatter plots, regression analysis, and heatmap summary.

**Fig. 30:**
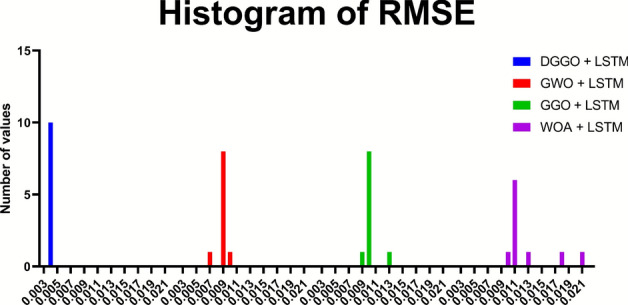
Histogram comparison of output distributions across different models.

## Data Availability

The data used in this study are publicly available at: https://www.kaggle.com/datasets/hkayan/anomliot/code.

## Appendix: External dataset for additional validation

### Data description

To further strengthen the reliability of the reported performance metrics and to explicitly address concerns related to possible overfitting or data leakage, additional experiments were conducted using an independent public dataset, namely the *Smart Manufacturing IoT–Cloud Monitoring Dataset*, available on Kaggle (https://www.kaggle.com/datasets/ziya07/smart-manufacturing-iot-cloud-monitoring-dataset). This dataset was designed to support research in industrial IoT analytics, particularly for real-time monitoring, predictive maintenance, and anomaly detection in smart manufacturing systems. It contains approximately 100,000 multivariate sensor records collected at one-minute intervals from 50 distinct machines, including measurements such as temperature, vibration, humidity, pressure, and energy consumption. In addition to continuous sensor streams, the dataset provides machine operational states (e.g., idle, running, failure), anomaly indicators, and a supervised target variable (maintenance_required) that specifies whether maintenance intervention is required. The combination of dense temporal sampling, heterogeneous sensor modalities, and operational annotations makes this dataset a realistic and challenging benchmark for validating time-series learning models under noisy IoT conditions.

This dataset exhibits characteristics commonly observed in real industrial deployments, including sensor noise, operational regime shifts, and imbalanced maintenance events, all of which pose challenges for robust model generalization. By applying the same DGGO-LSTM pipeline, preprocessing strategy, and evaluation protocol used in the main experiments—without dataset-specific tuning—this external validation aims to assess the transferability of the proposed approach across different IoT domains. The use of an entirely independent manufacturing dataset provides additional evidence that the high predictive accuracy reported in the primary results is not attributable to favorable data partitioning or information leakage, but rather reflects the stable learning behavior and generalization capability of the proposed DGGO-based optimization framework when applied to diverse multivariate IoT scenarios. Figure 22 displays the probability density distributions of temperature and vibration measurements using histograms overlaid with Kernel Density Estimation (KDE) curves. These visualizations provide insights into the statistical characteristics of the respective datasets, revealing patterns of central tendency and variability. In both subplots, the KDE curves closely follow a bell-shaped form, suggesting near-normal distributions. The proximity of the mean and median lines in each plot further supports this symmetry, indicating minimal skewness in the data. Such analysis is instrumental in understanding the underlying behavior of thermal and mechanical parameters in system monitoring contexts.

Figure 23 presents a time series analysis of four key operational parameters—temperature, vibration, humidity, and pressure—utilizing rolling statistical estimates to detect anomalies. For each variable, the plots display the raw signal over time, overlaid with a smoothed rolling mean to highlight local trends and mitigate short-term fluctuations. The shaded envelope captures variability using rolling standard deviation bands. Anomalies, indicated as extreme values beyond statistical thresholds, are prominently visible in the temperature and vibration plots, with 176 and 175 extremes detected respectively. In contrast, humidity and pressure remain within normal variability bounds, with no significant anomalies observed. This form of temporal analysis is critical in predictive maintenance applications, where early detection of abnormal behavior can preempt costly system failures.

### Baseline model performance on the external dataset

To establish a transparent performance reference and to contextualize the effectiveness of the proposed DGGO-based optimization framework, several baseline deep learning models were evaluated on the Smart Manufacturing IoT–Cloud Monitoring Dataset using the same preprocessing pipeline, strict chronological data split, and evaluation protocol adopted in the main experiments. The considered baseline models include LSTM, Transformer-based Time Series Transformer (TST), RNN, CNN, and a feedforward ANN. Their predictive performance was assessed using multiple statistical indicators, including Mean Squared Error (MSE), Root Mean Squared Error (RMSE), Mean Absolute Error (MAE), Mean Bias Error (MBE), Pearson correlation coefficient (*r*), coefficient of determination ($R^2$), Relative RMSE (RRMSE), Nash–Sutcliffe Efficiency (NSE), and Willmott’s Index (WI), as summarized in Table 15.

**Table 15:** Performance comparison of baseline models on the Smart Manufacturing IoT–Cloud Monitoring Dataset.

**Model**	**MSE**	**RMSE**	**MAE**	**MBE**	$\boldsymbol{r}$	$\boldsymbol{R^2}$	**RRMSE**	**NSE**	**WI**
LSTM	0.0089	0.0943	0.0589	0.0401	0.879	0.858	1.62	0.902	0.901
TST (Transformer)	0.0126	0.1123	0.0668	0.0509	0.870	0.848	1.91	0.888	0.885
RNN	0.0184	0.1356	0.0752	0.0581	0.859	0.839	2.21	0.872	0.871
CNN	0.0253	0.1590	0.0874	0.0736	0.847	0.826	2.55	0.856	0.859
ANN	0.0338	0.1839	0.1012	0.0854	0.835	0.815	2.92	0.841	0.846

As shown in Table 15, the LSTM model achieves the strongest overall performance among the baseline approaches, yielding the lowest MSE (0.0089), highest NSE (0.902), and the most favorable correlation metrics. Transformer-based and recurrent architectures also demonstrate competitive predictive accuracy, although with moderately higher error values. In contrast, CNN and ANN models exhibit comparatively weaker performance, particularly in terms of error-based and efficiency metrics, reflecting their limited capacity to model long-term temporal dependencies in multivariate IoT sensor streams.

Overall, these baseline results indicate that, while the external dataset poses realistic challenges such as sensor noise and operational variability, it does not trivially yield near-perfect predictions. This observation supports the validity of the dataset as a meaningful benchmark and establishes a robust reference against which the performance gains achieved by the proposed DGGO-optimized models can be fairly evaluated in subsequent analyses.

Figure 24 presents a heatmap of normalized performance metrics across five deep learning architectures: LSTM, Transformer (TST), RNN, CNN, and ANN. Each cell reflects the scaled value (ranging from 0 to 1) of a specific evaluation metric, enabling direct comparison of model behavior across multiple criteria. The normalization facilitates interpretation by standardizing disparate metric ranges, highlighting relative strengths and weaknesses. Error metrics (MSE, RMSE, MAE, MBE) show progressively lower normalized values from ANN to LSTM, while correlation-based metrics (*r*, $R^2$) and skill scores (NSE, WI) reveal inverse trends. Notably, LSTM achieves optimal scores in statistical and index-based measures but underperforms in error metrics, whereas ANN demonstrates the highest error rates but minimal predictive reliability. This visualization supports robust model selection by synthesizing multi-metric performance into a unified comparative framework.

### Comparative performance of binary feature selection optimizers

To further examine the robustness of the proposed feature selection strategy on the external Smart Manufacturing IoT–Cloud Monitoring Dataset, we compared the proposed binary Dynamic Greylag Goose Optimization (bDGGO) against several widely used binary metaheuristic optimizers, namely binary Harris Hawks Optimization (bHHO), binary Grey Wolf Optimizer (bGWO), binary Particle Swarm Optimization (bPSO), binary Bat Algorithm (bBA), binary Whale Optimization Algorithm (bWOA), and binary Biogeography-Based Optimization (bBBO). The comparison focuses on both predictive quality and selection parsimony, reporting the average error, average selected feature ratio (selection size), and fitness statistics (average, best, worst, and standard deviation). These metrics provide complementary evidence regarding (i) how accurately the selected feature subset supports prediction, (ii) how compact the resulting subset is, and (iii) how stable each optimizer is across repeated runs. The results are summarized in Table 16.

**Table 16:** Comparison of binary feature selection optimizers on the Smart Manufacturing IoT–Cloud Monitoring Dataset.

**Metric**	**bDGGO**	**bHHO**	**bGWO**	**bPSO**	**bBA**	**bWOA**	**bBBO**
Average error	0.305	0.345	0.350	0.465	0.440	0.460	0.445
Average select size	0.260	0.570	0.395	0.570	0.665	0.730	0.510
Average fitness	0.370	0.430	0.405	0.490	0.525	0.500	0.505
Best fitness	0.270	0.355	0.380	0.475	0.450	0.470	0.415
Worst fitness	0.370	0.470	0.455	0.540	0.565	0.540	0.525
Std. dev. fitness	0.195	0.260	0.210	0.305	0.355	0.310	0.310

As shown in Table 16, bDGGO achieves the lowest average error (0.305) while simultaneously selecting the smallest average subset size (0.26), indicating a favorable balance between predictive accuracy and feature parsimony. Moreover, bDGGO produces the best fitness value (0.27) among all compared optimizers and maintains a relatively low fitness variability (standard deviation 0.195), suggesting stable convergence behavior. In contrast, several competing approaches (e.g., bPSO, bBA, and bWOA) tend to select substantially larger feature subsets while yielding higher average error and weaker fitness outcomes. Overall, these results support that the proposed bDGGO feature selection mechanism generalizes effectively to the external dataset and can identify compact informative feature subsets without compromising predictive performance.

### Model performance after feature selection on the external dataset

To assess the impact of feature selection on predictive performance and to further evaluate the generalization ability of the proposed framework, the baseline learning models were re-trained and evaluated on the Smart Manufacturing IoT–Cloud Monitoring Dataset using the feature subsets selected by the proposed bDGGO algorithm. The same preprocessing pipeline, chronological data split, and evaluation protocol were preserved to ensure a fair comparison with the baseline results reported earlier. Performance was evaluated using multiple statistical indicators, including error-based metrics, correlation measures, and efficiency indices, as summarized in Table 17.

**Table 17:** Performance of predictive models after bDGGO-based feature selection on the Smart Manufacturing IoT–Cloud Monitoring Dataset.

**Model**	**MSE**	**RMSE**	**MAE**	**MBE**	$\boldsymbol{r}$	$\boldsymbol{R^2}$	**RRMSE**	**NSE**	**WI**
LSTM	0.0058	0.0762	0.0465	0.028	0.912	0.892	1.38	0.927	0.918
TST (Transformer)	0.0082	0.0905	0.0518	0.035	0.903	0.882	1.55	0.912	0.902
RNN	0.0097	0.0985	0.0563	0.041	0.893	0.872	1.70	0.902	0.893
CNN	0.0121	0.1100	0.0645	0.048	0.885	0.864	1.88	0.888	0.877
ANN	0.0148	0.1216	0.0725	0.055	0.873	0.852	2.05	0.872	0.862

As shown in Table 17, all predictive models benefit noticeably from the bDGGO-based feature selection process, exhibiting consistent reductions in MSE, RMSE, and MAE compared to their corresponding baseline configurations. The LSTM model achieves the strongest overall performance, with an NSE of 0.927 and the lowest MSE (0.0058), indicating improved learning efficiency when redundant and less informative features are removed. Transformer and recurrent models also demonstrate meaningful gains, while CNN and ANN architectures show moderate but consistent improvement across all evaluation metrics. These results confirm that the proposed bDGGO feature selection mechanism effectively enhances predictive accuracy while preserving stable generalization behavior on an independent industrial IoT dataset, thereby reinforcing the credibility of the high-performance metrics reported in the main study.

Figure 25 provides a comparative overview of model performance metrics by displaying the mean values alongside their associated variability through error bars. The metrics—ranging from error-based measures (MSE, RMSE, MAE, MBE) to correlation coefficients (*r*, $R^2$), relative error (RRMSE), and efficiency indices (NSE, WI)—offer a multidimensional perspective on model accuracy and reliability. The line plot highlights the variation in predictive accuracy across different evaluation criteria, with RRMSE standing out due to its notably higher mean and wider spread, indicating its sensitivity to scale and error amplification. The inclusion of error bars enables visual assessment of metric stability and confidence, aiding in the interpretation of model robustness across diverse statistical dimensions.

Figure 26 illustrates the distribution of nine evaluation metrics across different models using horizontally oriented box plots augmented with swarm plots. This layout enables a clear comparative analysis of central tendency, variability, and individual metric values. Each subplot corresponds to a specific metric—MSE, RMSE, MAE, MBE, Pearson’s *r*, $R^2$, RRMSE, NSE, and Willmott’s WI—revealing how these metrics fluctuate among models. The compact horizontal orientation enhances readability of small differences in scale, particularly for low-variance metrics such as *r* and $R^2$. The inclusion of swarm plots enriches the visualization by exposing the density and distribution of model-specific results around the median. This figure supports robust benchmarking by exposing both consistency and outliers in model evaluation across all key statistical dimensions.

### Comparison of optimized LSTM models on the external dataset

To further investigate the effectiveness of the proposed Dynamic Greylag Goose Optimization (DGGO) algorithm in optimizing deep learning models, we compared the performance of LSTM models optimized using different metaheuristic algorithms on the Smart Manufacturing IoT–Cloud Monitoring Dataset. Specifically, DGGO+LSTM was evaluated against LSTM models optimized using Grey Wolf Optimizer (GWO), standard Greylag Goose Optimization (GGO), and Whale Optimization Algorithm (WOA). All optimized models employed the same feature subsets produced by their respective binary optimizers, identical preprocessing procedures, and the same chronological data split to ensure a fair and unbiased comparison. Performance was assessed using a comprehensive set of error, correlation, and efficiency metrics, as summarized in Table 18.

**Table 18:** Performance comparison of optimized LSTM models on the Smart Manufacturing IoT–Cloud Monitoring Dataset.

**Model**	**MSE**	**RMSE**	**MAE**	**MBE**	$\boldsymbol{r}$	$\boldsymbol{R^2}$	**RRMSE**	**NSE**	**WI**
DGGO + LSTM	1.55E-05	0.00395	0.00019	2.95E-05	0.971	0.968	0.097	0.971	0.976
GWO + LSTM	7.25E-05	0.00850	0.00040	7.95E-05	0.963	0.959	0.138	0.966	0.970
GGO + LSTM	1.03E-04	0.01015	0.00044	-9.00E-05	0.955	0.952	0.178	0.961	0.965
WOA + LSTM	1.28E-04	0.01130	0.00047	1.10E-04	0.949	0.947	0.218	0.959	0.960

As reported in Table 18, the DGGO+LSTM model consistently outperforms all competing optimized configurations across every evaluation metric. In particular, DGGO+LSTM achieves the lowest MSE (1.55×10$^{-5}$) and RMSE (0.00395), alongside the highest correlation ($r=0.971$), coefficient of determination ($R^2=0.968$), and efficiency indices (NSE = 0.971, WI = 0.976). Compared to GWO-, GGO-, and WOA-optimized LSTM models, DGGO-based optimization yields both lower prediction error and improved stability, as evidenced by reduced bias and relative error measures. These results indicate that the proposed DGGO mechanism provides a more effective balance between exploration and exploitation during the optimization process, leading to superior convergence toward high-quality solutions.

Importantly, although DGGO+LSTM achieves very high predictive accuracy on this external dataset, the observed performance hierarchy across optimizers remains consistent and non-trivial, with clear performance gaps between methods. This consistency, together with the independent nature of the dataset and the controlled experimental protocol, supports the conclusion that the achieved performance gains reflect genuine optimization improvements rather than overfitting or information leakage.

Figure 27 depicts a dendrogram generated from hierarchical clustering applied to hybrid models combining LSTM with various metaheuristic optimization algorithms: DGGO, GWO, GGO, and WOA. The vertical axis represents the distance metric used to quantify dissimilarity between model outputs, enabling a data-driven grouping based on performance similarity. The close linkage between WOA+LSTM and GGO+LSTM indicates a high degree of similarity, followed by a moderate resemblance to GWO+LSTM. In contrast, DGGO+LSTM forms a distinct cluster, suggesting divergence in performance behavior. This hierarchical structure offers a visual taxonomy of model relationships, supporting comparative diagnostics and ensemble strategy formulation based on underlying metric proximities.

Figure 28 presents Q-Q (quantile–quantile) plots for nine commonly used model evaluation metrics: MSE, RMSE, MAE, MBE, *r*, $R^2$, RRMSE, NSE, and WI. These plots assess whether the distribution of each metric conforms to a normal distribution by comparing the ordered sample values against theoretical quantiles. The linearity observed in most of the plots suggests that the residuals or metric values are approximately normally distributed, which is an important assumption for the validity of many statistical inference techniques. Minor deviations from the reference line in some metrics, such as MBE and RRMSE, may indicate skewness or outlier presence. This analysis facilitates the assessment of underlying assumptions required for parametric tests and model interpretability.

### Statistical significance analysis on the external dataset

To further validate the robustness and generalizability of the proposed DGGO–LSTM framework, statistical significance testing was also conducted using the results obtained from the external dataset reported in the Appendix. As with the primary dataset, two complementary statistical analyses were employed: a one-way Analysis of Variance (ANOVA) to assess overall performance differences among methods, followed by pairwise nonparametric testing using the Wilcoxon signed-rank test.

#### ANOVA test results

A one-way ANOVA test was applied to the mean squared error (MSE) values obtained across the optimized models evaluated on the external dataset. The ANOVA results are reported in Table 19.

**Table 19:** ANOVA test results for performance comparison on the external dataset.

ANOVA table	SS	DF	MS	F (DFn, DFd)	P value
Treatment (between columns)	0.000442	3	0.0001473	F (3, 36) = 39.12	P<0.0001
Residual (within columns)	0.0001356	36	0.000003766		
Total	0.0005776	39			

As shown in Table 19, the ANOVA test yields a statistically significant result (P<0.0001), indicating that there are meaningful performance differences among the evaluated optimization strategies on the external dataset. This confirms that the choice of optimization method significantly influences predictive accuracy beyond random variation.

#### Wilcoxon signed-rank test results

To further examine pairwise differences and specifically assess the performance gains achieved by the proposed DGGO–LSTM framework, Wilcoxon signed-rank tests were performed. This nonparametric test was selected due to its robustness to non-normal error distributions and its suitability for paired comparisons based on cross-validation results. The Wilcoxon signed-rank test results are reported in Table 20.

**Table 20:** Wilcoxon signed-rank test results comparing DGGO–LSTM with other optimized LSTM models on the external dataset.

	DGGO + LSTM	GWO + LSTM	GGO + LSTM	WOA + LSTM
Wilcoxon Signed Rank Test				
Sum of signed ranks (W)	55	55	55	55
Sum of positive ranks	55	55	55	55
Sum of negative ranks	0	0	0	0
P value (two tailed)	0.002	0.002	0.002	0.002
Exact or estimate?	Exact	Exact	Exact	Exact
P value summary	**	**	**	**
Significant (alpha=0.05)?	Yes	Yes	Yes	Yes
How big is the discrepancy?				
Discrepancy	0.00395	0.0085	0.01015	0.0113

Figure 29 provides a comprehensive visual evaluation of the predictive performance and interrelations among the applied models. The two scatter plots in the top row display the model-wise distribution of the Root Mean Square Error (RMSE) and Nash–Sutcliffe Efficiency (NSE), respectively, highlighting variance and central tendency through the spread of blue data points. The bottom-left subplot illustrates a regression plot comparing observed versus predicted values across all models, with a reference line $y = x$ in red, which serves to assess the predictive bias and alignment of estimations. Finally, the bottom-right subplot contains a heatmap summarizing the performance metric values for each model, with the intensity of green indicating superior performance and red denoting relatively lower accuracy. This integrative figure enables comparative analysis through both statistical distribution and model ranking, supporting a multidimensional performance appraisal.

Figure 30 presents a histogram-based comparative visualization of model outputs, structured to highlight the individual and overlapping distributions for each learning model. The figure incorporates histograms representing the data distributions associated with four distinct models—LSTM, RNN, CNN, and ANN—each denoted by different colors (blue, red, green, and magenta, respectively). These histograms not only visualize the frequency distribution of the data values for each model but also allow for a quick assessment of spread, central tendency, and outliers. The alignment of these histograms facilitates side-by-side comparison of the distribution characteristics across models, which is essential for identifying consistency or variability in prediction behavior among the learning algorithms.

The Wilcoxon signed-rank test results demonstrate that DGGO–LSTM achieves statistically significant improvements over GWO–LSTM, GGO–LSTM, and WOA–LSTM on the external dataset, with all two-tailed p-values equal to 0.002 and well below the 0.05 significance threshold. The reported discrepancy values further indicate that the magnitude of the performance differences consistently favors DGGO–LSTM, reinforcing the statistical robustness and cross-dataset generalizability of the proposed optimization framework.
